# *eIF4B* and *eIF4H* mediate GR production from expanded G4C2 in a *Drosophila* model for *C9orf72*-associated ALS

**DOI:** 10.1186/s40478-019-0711-9

**Published:** 2019-04-25

**Authors:** Lindsey D. Goodman, Mercedes Prudencio, Ananth R. Srinivasan, Olivia M. Rifai, Virginia M.-Y. Lee, Leonard Petrucelli, Nancy M. Bonini

**Affiliations:** 1 Neuroscience Graduate Group, Perelman School of Medicine, University of Pennsylvania, Philadelphia, PA 19104 USA; 2 Department of Biology, University of Pennsylvania, Philadelphia, PA 19104 USA; 3 Department of Neuroscience, Mayo Clinic, Jacksonville, FL 32224 USA; 4 Center for Neurodegenerative Disease Research, Perelman School of Medicine, University of Pennsylvania, Philadelphia, PA 19104 USA

**Keywords:** *eIF4B*, *eIF4H*, *Drosophila*, Amyotrophic lateral sclerosis (ALS) (Lou Gehrig disease), *C9orf72*, RAN-translation, Neurodegeneration

## Abstract

The discovery of an expanded (GGGGCC)n repeat (termed G4C2) within the first intron of *C9orf72* in familial ALS/FTD has led to a number of studies showing that the aberrant expression of G4C2 RNA can produce toxic dipeptides through repeat-associated non-AUG (RAN-) translation. To reveal canonical translation factors that impact this process, an unbiased loss-of-function screen was performed in a G4C2 fly model that maintained the upstream intronic sequence of the human gene and contained a GFP tag in the GR reading frame. 11 of 48 translation factors were identified that impact production of the GR-GFP protein. Further investigations into two of these, *eIF4B* and *eIF4H*, revealed that downregulation of these factors reduced toxicity caused by the expression of expanded G4C2 and reduced production of toxic GR dipeptides from G4C2 transcripts. In patient-derived cells and in post-mortem tissue from ALS/FTD patients, *eIF4H* was found to be downregulated in cases harboring the G4C2 mutation compared to patients lacking the mutation and healthy individuals. Overall, these data define *eIF4B* and *eIF4H* as disease modifiers whose activity is important for RAN-translation of the GR peptide from G4C2-transcripts.

**Electronic supplementary material:**

The online version of this article (10.1186/s40478-019-0711-9) contains supplementary material, which is available to authorized users.

## Introduction

In Amyotrophic Lateral Sclerosis (ALS) and Frontotemporal Degeneration (FTD), the presence of a hexanucleotide expansion of > 30 GGGGCC repeats (termed G4C2) within the *C9orf72* gene is the most prominent mutation in familial disease [17, 65]. The mechanisms underlying potential toxicity associated with G4C2 are still being defined with two leading hypotheses centering around gain-of-function mechanisms [5, 93]: sequestration of RNA-binding proteins by the aberrant expression of sense- and antisense- G4C2 RNA [30, 90]; repeat-associated non-AUG (RAN-) translation of repeat-containing transcripts produce dipeptides that are toxic to neurons [4, 25, 47–49, 56, 58]. Five dipeptides can be produced from these transcripts, depending on the reading frame: GA and GR (sense strand associated), PA and PR (antisense strand associated), and GP (produced from both sense and antisense strands).

In recent years, it has become clear that dipeptides produced from G4C2 RNA transcripts cause neurodegenerative effects [5, 93]. Of the 5 potential RAN-translation products, GR and PR cause particularly strong degenerative phenotypes in multiple model systems, including *Drosophila* [22, 53]. Therefore, increasing understanding of the mechanisms underlying expression of these dipeptides would highlight potential therapeutic avenues centered around preventing their expression.

Many mechanistic questions remain regarding RAN-translation in G4C2-associated disease. Recent investigations have drawn a number of parallels between mechanisms underlying general translation [10, 77, 78] and RAN-translation [37, 96], finding that dipeptide production is sensitive to the inhibition/downregulation of canonical translation factors: eIF4E, eIF4G, eIF4A, eIF2α, eIF2A [12, 27, 84, 97]. Of interest, eIF4A is a DEAD-Box helicase [3], and thus may be important for the unwinding of G4C2-RNA for translation. While eIF4A has relative weak helicase activity alone, this can be significantly stimulated by accessory proteins eIF4B and eIF4H [24, 31, 59, 68, 70, 74, 82, 91]. These latter factors contain RRM-domains and, importantly, have been reported to interact directly with the G4C2 RNA [14, 29, 72].

In an unbiased, directed screen for canonical translation factors, we identified 11 potential translation factors that modulate GR-production in G4C2-expressing flies. Further investigations into two of these, *eIF4B* and *eIF4H1* (fly orthologue to *eIF4H*), further defined them as modifiers of G4C2-toxicity. Their downregulation significantly reduced GR-levels in animals expressing the repeat. Further investigations into eIF4B and eIF4H in C9+ derived cells revealed that eIF4H was significantly downregulated. *eIF4H* downregulation also occurred in post-mortem tissue from C9+ ALS/FTD compared to C9- ALS/FTD and healthy individuals. This work identifies eIF4B and eIF4H as important disease modifiers that alter RAN-translation of the GR-reading frame.

## Results

### GFP-tagged GR dipeptides are produced in LDS-(G4C2)_n_ flies with expanded (> 30) repeats

**Fig. 1:**
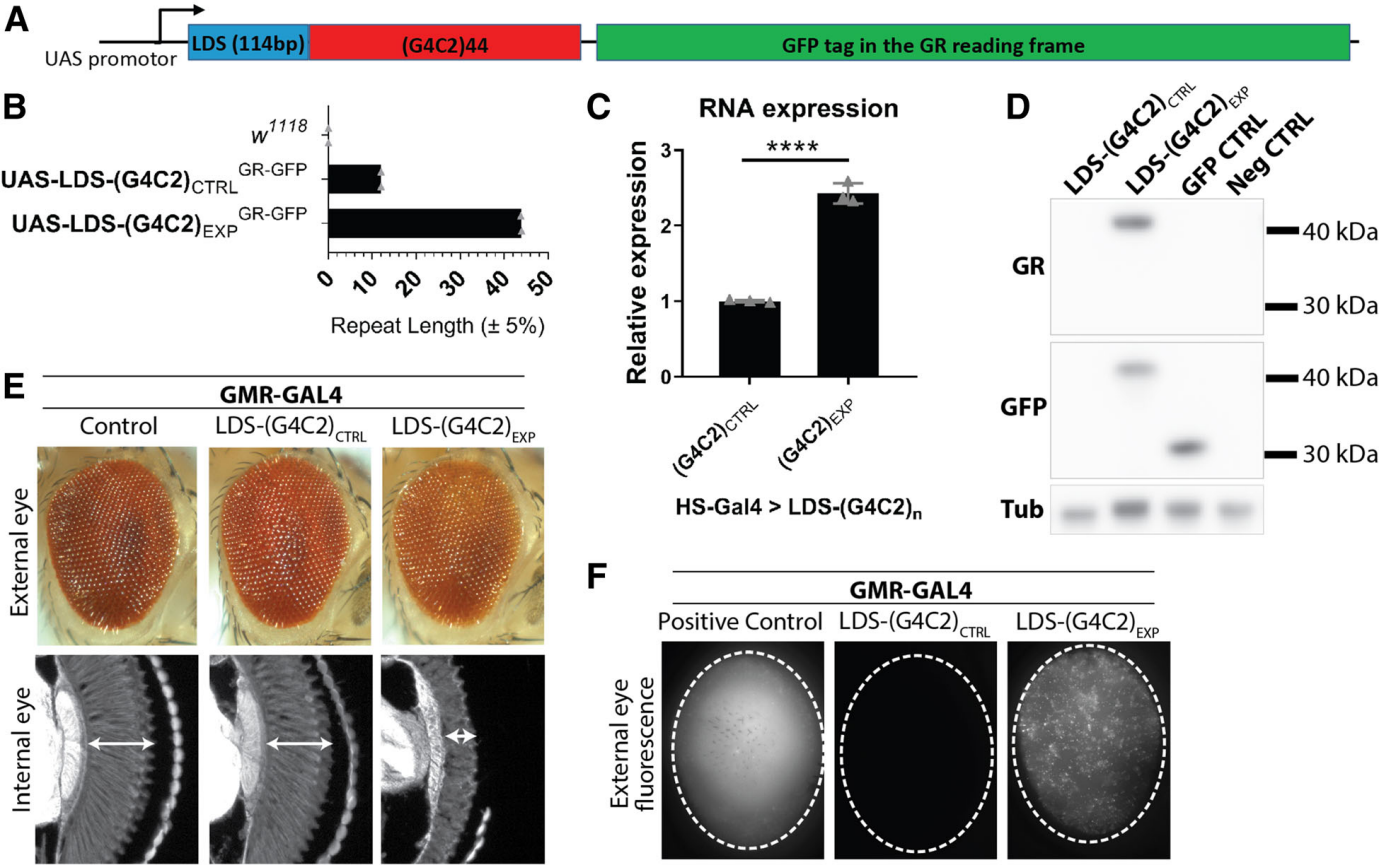
Expanded G4C2 transgenes produce GFP-tagged GR. **a**. A new transgenic (G4C2)n model was developed to look at RAN-translation of the GR reading frame. A “leader” sequence (LDS) was added 5′ of the repeat: 114 bp of intronic sequence found upstream of the repeat in patient samples. The GR reading frame has an in-frame GFP coding sequence 3′ of the repeat that lacks an ATG initiation. **b**. To define the number of repeats inserted into genomes of *w*^*1118*^ transgenic flies, PCR reactions were developed that amplified the repeat and its flanking region. The number of repeats were calculated from PCR product lengths measured on a Bioanalyzer and agarose gel. Shown: the maximum number of repeats found in the control (CTRL) or expanded (EXP) G4C2 fly lines; individual data points from 2 independent DNA preps with mean. **c**. qPCR analysis for RNA levels between control and expanded G4C2 fly lines. Shown: individual data points with mean ± SD. Statistics: unpaired student t-test, *p*-value **** < 0.0001. **d**. Western immunoblots confirmed GR is produced and successfully tagged with GFP in EXP-G4C2 flies. No GR/GFP was detected in control G4C2 flies, even with overexposure (Additional file 1: Figure S1). Uncropped westerns (Additional file 1: Figure S4) **e**. External and internal eye analysis in animals expressing G4C2 or control (DSRED) transgenes using GMR-GAL4. Degeneration was seen only in LDS-(G4C2)_EXP_ animals: external pigment loss and reduced integrity of internal retina tissue. **f**. External eye imaging for fluorescence caused by transgene expression. Positive (DSRED) control flies show uniform, diffuse signal. Control G4C2 flies show no signal, even with increased exposure (data not shown). Expanded G4C2 flies show GFP puncta. Shown (**e**-**f**): representative images while all conditions were tested 2+ times. For full genotypes see Additional file 7: Table S1

We previously identified a number of translation factors as modifiers of G4C2-toxicity [26]. To investigate these and other factors in the context of RAN-translation, a new *C9orf72* fly model for ALS/FTD was designed (Fig. 1a). This model contained the 114-base pair sequence immediately upstream of the repeat in intron 1 of *C9orf72* in ALS/FTD patient genomes (termed a “leader” sequence; LDS). The addition of this sequence puts the repeat in a more patient-relevant context while this region is likely to influence pathological mechanisms, including RAN-translation [36, 73, 87, 96]. G4C2 expansions can produce three sense-strand associated dipeptides: GA, GR, and GP. Importantly, of these GR is associated with extreme toxicity in multiple models, including flies [22, 53]. To facilitate investigations into genes that may impact RAN-translation of GR, a GFP tag (lacking an ATG initiation codon) was added 3′-prime of the repeat in the GR-reading frame.

LDS-G4C2 transgenes were randomly inserted into the genome of *w*^*1118*^ animals. To define the number of repeats inserted into individual fly lines, primers that flanked the G4C2 repeat were used to PCR amplify the region [26]. The number of repeats was then calculated from the length of the PCR products, resolved by agarose gel electrophoresis and Bioanalyzer. Two primary fly transgenic lines were defined: a control (CTRL) line containing short (G4C2)_≤12_ repeats and an expanded (EXP) line containing (G4C2)_≤44_ repeats (Fig. 1b). By quantitative-real time PCR (qPCR), these two lines expressed significantly different G4C2 RNA levels (Fig. 1c), most likely the result of variability in insertion site within the fly genome [43].

Western immunoblots were used to determine whether a (GR)n dipeptide was produced from the LDS-G4C2 transgenes, despite the absence of an AUG-start codon in the GR-reading frame. LDS-G4C2 transgenes were expressed in the fly eye using GMR-GAL4 and protein lysates were prepared from heads. Using an antibody designed to target the GR-dipeptide [46], we found that a GR peptide was produced only from the expanded LDS-(G4C2)_EXP_ fly line (Fig. 1d). Re-probing with an anti-GFP antibody confirmed that the GR-dipeptides produced were tagged with GFP. GFP expressing control flies confirmed that the molecular weight of the GR/GFP band in LDS-(G4C2)_EXP_ animals was higher than GFP alone. As LDS-(G4C2)_EXP_ lines had 2.5-fold higher RNA expression than the LDS-(G4C2)_CTRL_, longer exposure times were also evaluated and continued to show no GR/GFP signal in LDS-(G4C2)_CTRL_ expressing animals (Additional file 1: Figure S1).

To define potential toxicity associated with LDS-(G4C2)_EXP _, transgenes were expressed in the fly optic system using GMR-GAL4 (Fig. 1e). LDS-(G4C2)_CTRL_ animals showed external and internal eye morphologies similar to controls, supporting that the short repeat was not toxic [22, 26, 41, 53]. In contrast, expression of LDS-(G4C2)_EXP_ caused mild pigment loss externally and dramatic loss of retinal tissue internally, indicative of neurodegeneration.

To further assess GR production, fluorescence imaging of the fly eyes revealed that the LDS-(G4C2)_EXP_ expressing animals produced GFP-positive puncta (Fig. 1f). In contrast, a control fluorescence protein (DSRED) did not show puncta formation but rather had a uniform diffuse signal, indicating that the unique punctate fluorescence pattern seen with LDS-(G4C2)_EXP_ was the result of the GR. LDS-(G4C2)_CTRL_ animals were also imaged and showed no GFP signal, even with 5-10x longer exposure time (data not shown).

Overall, these data indicate that expression of LDS-(G4C2)_EXP_ in flies can induce toxicity and that GFP-tagged GR are produced by an expanded LDS-G4C2 transcript.

### A loss of function screen for candidate RAN-translation factors

**Fig. 2:**
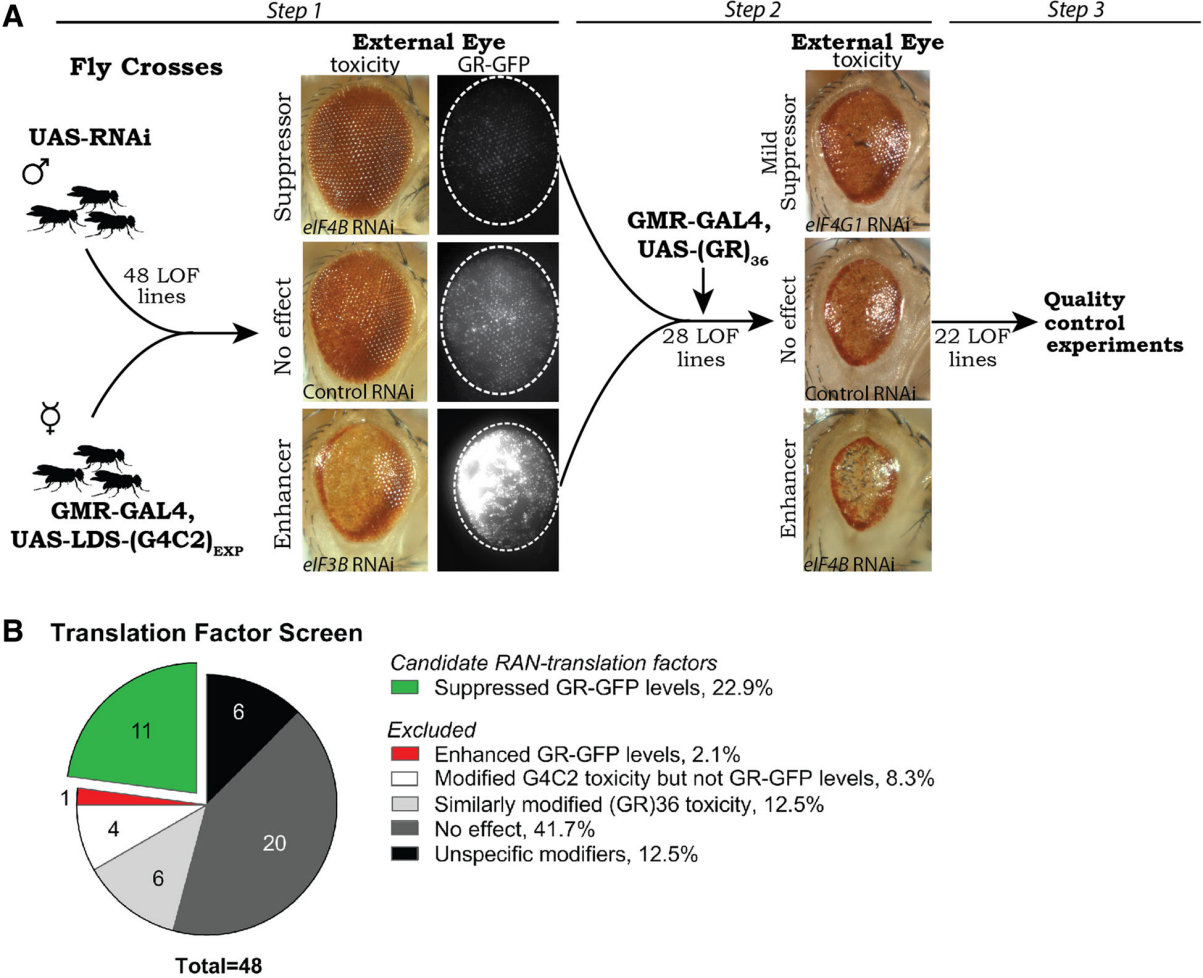
A screen of translation factors reveals those important for expression of GR from (G4C2)_EXP_. **a**. To identify canonical translation factors that may be involved in RAN translation of G4C2 in the GR reading frame, a loss-of-function (LOF) based screen was designed utilizing previously developed RNAi [57, 60] or LOF mutant fly lines [6, 7, 80, 81] targeting 48 of 56 (86%) known translation factors [50]. Individual translation factors were downregulated in animals expressing LDS-(G4C2)_EXP_ and any that altered the external eye phenotype and/or GR-GFP levels were defined *(Step 1)*. These 28 LOF lines were further tested in (GR)_36_ expressing animals, defining 6 that acted similarly on GR-associated toxicity *(Step 2)*. Additional quality control experiments excluded LOF lines that altered expression from a control (*LacZ*) transgene by western immunoblot and/or altered a WT eye morphology when expressed alone, as described [13, 26, 41] *(Step 3)*. **b**. Summary of screen results. Overall, 11 translation factors were identified as candidate RAN translation factors as their depletion reduced GR-GFP levels. Overall, excluded LOF lines either: altered toxicity of G4C2 flies but did not alter GR-GFP levels, similarly altered toxicity in a non-G4C2, GR fly model arguing that these acted downstream of GR production, had no effect on G4C2 toxicity or GR-GFP levels, or were “unspecific” modifiers identified by quality control experiments. Shown: representative images while all RNAi were tested 2+ times for effects under each condition. Details on LOF lines used and complete results with each line can be found in Additional file 2: Table S2. For full genotypes and RNAi lines see Additional file 7: Table S1

Despite recent advances into mechanisms underlying G4C2-associated RAN-translation, a full understanding of which canonical translation factors are involved remains unclear [12, 27, 37, 84, 96]. To define translation factors that may mediate GR-associated RAN-translation, we designed a loss-of-function (LOF) fly screen utilizing external eye imaging for toxicity, and GR-GFP fluorescence of the eyes for protein, in LDS-(G4C2)_EXP_ expressing animals (Fig. 2a). 48 RNAi [57, 60] or LOF mutant [6, 7, 80, 81] fly lines were obtained that target specific translation factors, covering 86% of the 56 known translation factors in the fly [50]. 40 of 48 (83.3%) lines were RNAi and 8 of 48 (16.6%) were mutant lines.

28 of the 48 tested LOF lines altered toxicity and/or GR-GFP levels caused by LDS-(G4C2)_EXP_ expression in the fly eye, assessed by comparing images with controls (Fig. 2a, *step 1*). These 28 lines were further examined in a fly model that expresses (GR)_36_ from a non-G4C2 transcript [53], to determine if they acted downstream of toxic GR-production in the LDS-(G4C2)_EXP_ animals (Fig. 2a, *step 2*). 6 of the LOF lines targeting translation factors were found to similarly alter GR-induced toxicity in this model and were not further studied. The remaining 22 LOF lines were further tested for unspecific effects using quality control experiments (Fig. 2a, *step 3*) [13, 26, 41, 55]. Specifically, lines were examined to eliminate those that cause an effect when expressed on their own in the eye and tested to confirm no effect on the protein levels of a control (*LacZ*) transgene.

In summary, 20 of the lines did not alter toxicity or GR-GFP levels in LDS-(G4C2)_EXP_ expressing animals (Fig. 2b). 17 lines were excluded from further study because they either caused increased GR-GFP levels (1 line), altered LDS-(G4C2)_EXP_ toxicity but not GR-GFP signal (4 lines), could alter GR-toxicity independent of G4C2-RNA (6 lines), or failed quality control experiments (6 lines; termed “unspecific modifiers”). Thus, from the screen of 48 factors, 11 candidate RAN-translation factors were identified (Table 1, Additional file 2: Table S2).

**Table 1:**
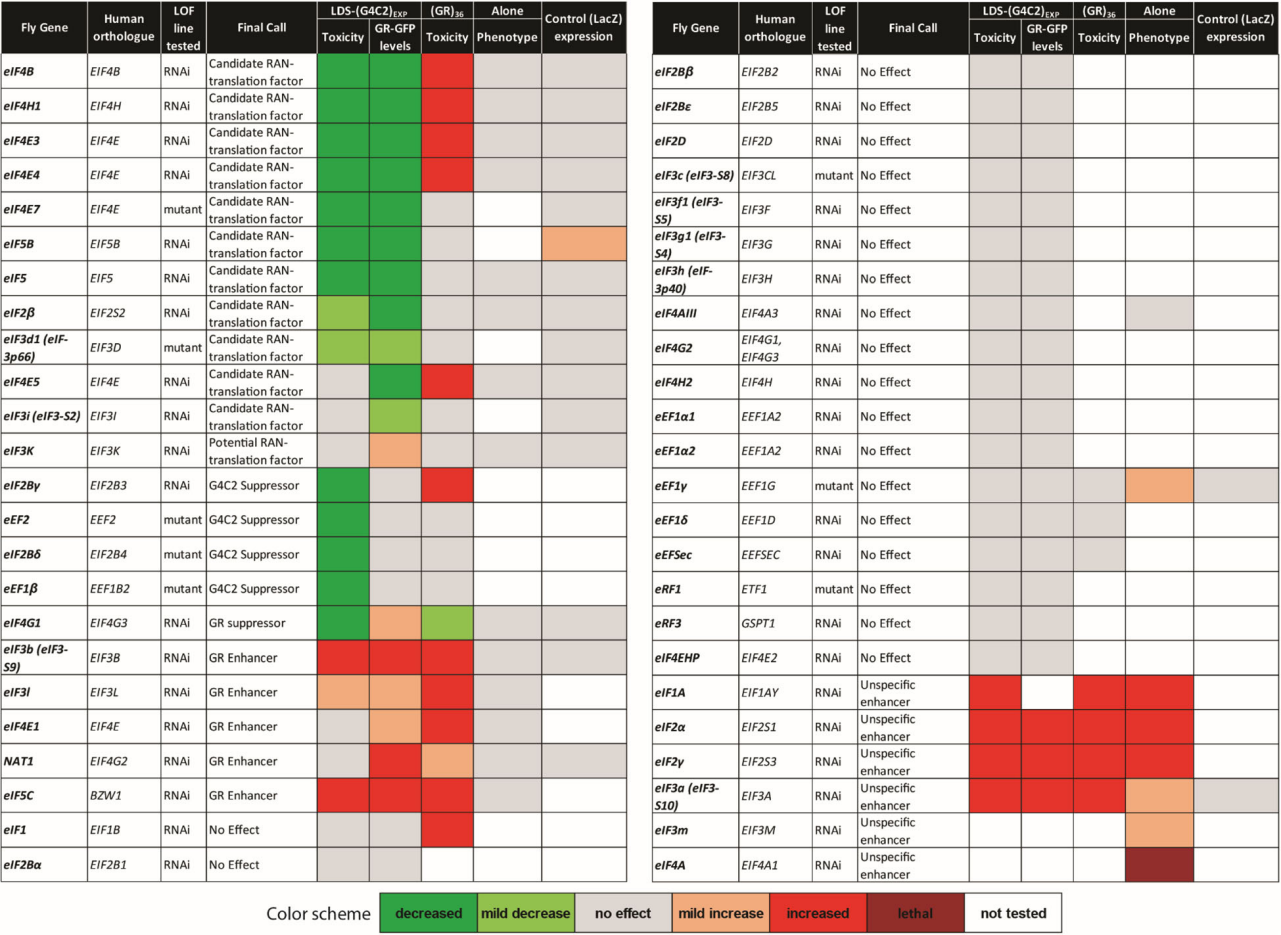
Translation factor screen results in LDS-(G4C2)_EXP_ expressing flies.

### Depletion of *eIF4B* or *eIF4H1* mitigates toxicity in LDS-(G4C2)_EXP_ animals

Of the 11 factors that reduced GR-GFP levels, eIF4B and eIF4H1 (fly orthologue to human eIF4H) were intriguing. These two factors have independent and redundant roles in activating eIF4A [24, 31, 59, 68, 70, 74, 82, 91] which was recently identified as a RAN-translation factor in a G4C2-model [27, 84]. Further, eIF4B and eIF4H had previously been reported to bind G4C2 RNA through RNA recognition motifs (RRMs) [14, 29, 72].

**Fig. 3:**
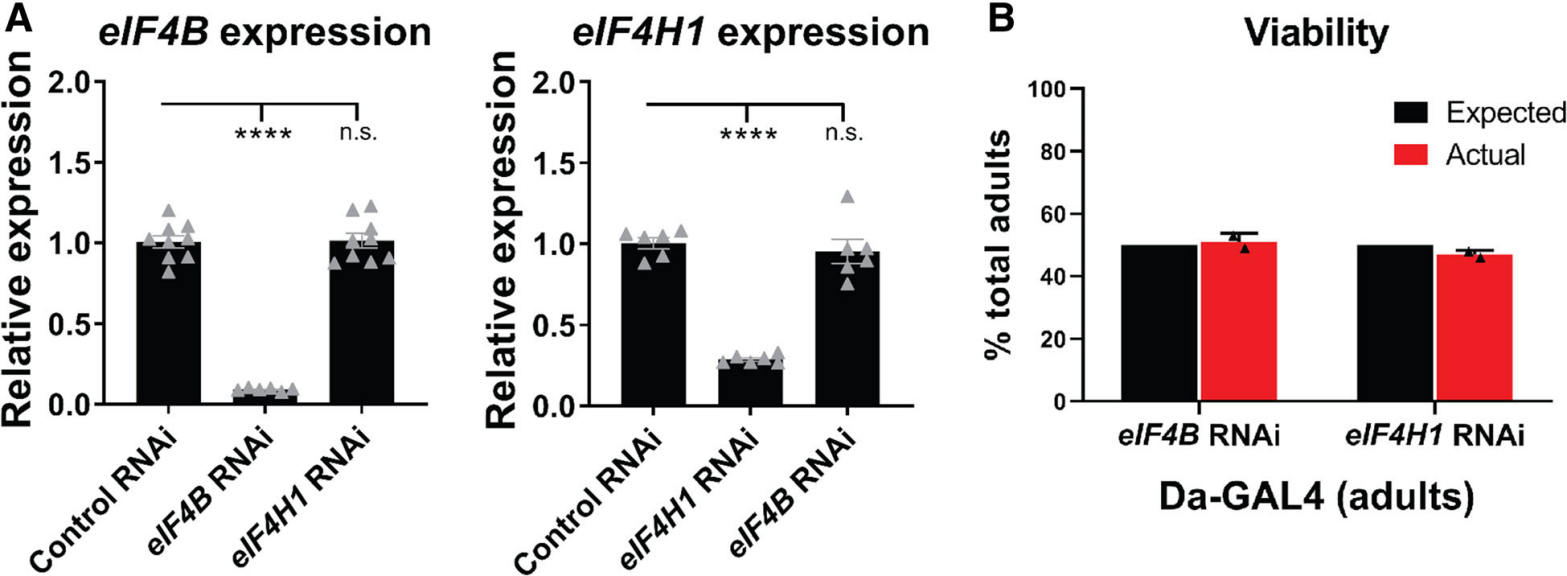
Analysis of eIF4B and eIF4H1 RNAi in control flies. **a**. RNA levels produced from *eIF4B* or *eIF4H1* were assessed by qPCR in flies ubiquitously expressing RNAi (by Daughterless-GAL4). Statistics: one-way ANOVAs with Tukey’s multiple comparison correction, *p*-values **** < 0.0001, *** < 0.001, ** < 0.01, * < 0.05, no significance > 0.05. Shown: individual data points from 2 independent experiments with mean ± SEM. **b**. Viability studies in *Drosophila* reveal that RNAi-depletion of eIF4B or eIF4H1 does not significantly alter the number of adult flies expected to eclose. Shown: ratio of progeny from two individual crosses with RNAi compared to sibling animals with the balancer chromosome that reach adulthood (1-2d adult animals). Comparing the # progeny that eclose from a single vial compensates for differences in mating variability, fertilized eggs laid, among other variables. However, we note that the presence of the balancer chromosome could potentially cause mild sub-viability to adulthood. Crosses: *RNAi/CyO* x *Da-GAL4 (III)*; counted progeny: *RNAi/+; Da-GAL4/+* and *CyO/+; Da-GAL4/+*. RNAi lines: control (JF01355), *eIF4B* RNAi (HMS04503), *eIF4H1* RNAi (HMS04504). For full genotypes see Additional file 7: Table S1

To further investigate *eIF4B* and *eIF4H1* as modifiers of LDS-(G4C2)_EXP_ in flies, a second, independent set of RNAi lines targeting these genes was obtained (termed RNAi-2). All RNAi lines were confirmed to downregulate the expected targets, *eIF4B* or *eIF4H1* (Fig. 3a, Additional file 1: Figure S3A). Further, *eIF4B* RNAi did not cause reduced expression of *eIF4H1*, and vice versa, indicating that expression of these two genes is independent and that the RNAi lines are specific. Interestingly, ubiquitous downregulation of *eIF4B* or *eIF4H1* by RNAi produced viable adults with no obvious phenotype (Fig. 3b), supporting that these genes are not essential in the fly (also [33]).

**Fig. 4:**
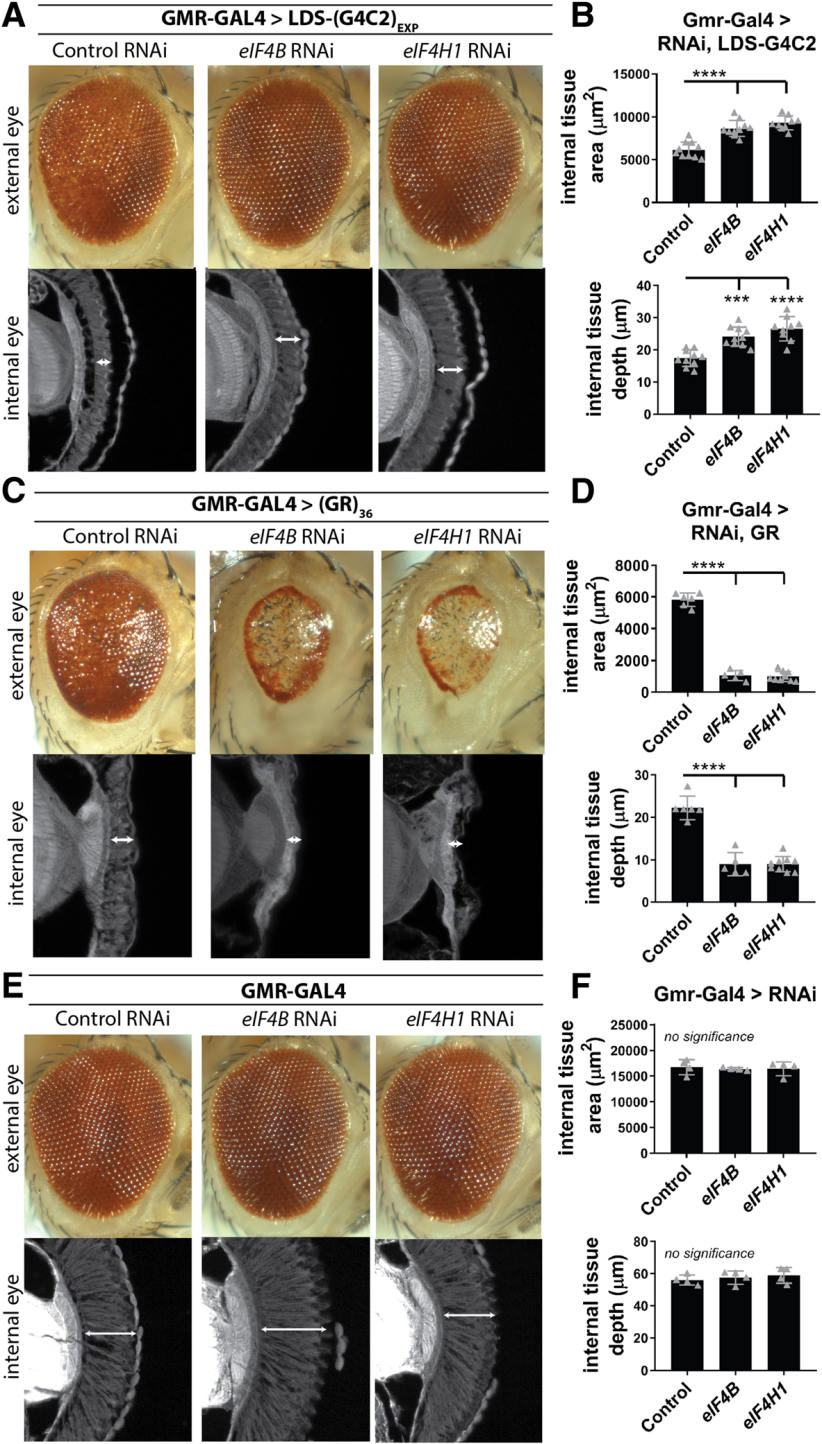
Depletion of *eIF4B* and *eIF4H1* selectively suppresses LDS-(G4C2)_EXP_ associated toxicity. **a**. Using GMR-GAL4, RNAi-mediated depletion of *eIF4B* or *eIF4H1* in LDS-(G4C2)_EXP_ expressing flies results in reduced toxicity in both the external and internal eye: seen externally by recovered pigment and ommatidial structure, seen internally by recovered retinal tissue integrity. **b**. Blinded quantification of internal retina tissue was done by measuring the total surface area of tissue present and by measuring the depth of the tissue at the position where the optic chiasm occurs. *n* = 9–10 animals per genotype. **c**. *eIF4B* or *eIF4H1* RNAi was expressed in (GR)36 flies (GMR-GAL4) and effects on GR-associated toxicity were observed in the external and internal eye. **d**. Blinded quantification of internal retina tissue. *n* = 5–9 animals per genotype. **e**. *eIF4B* or *eIF4H1* RNAi was expressed in control flies (GMR-GAL4) and effects on the normal eye were observed externally and internally. **f**. Blinded quantification of internal retina tissue. *n* = 4 animals per genotype. For graphs, shown are individual data points representing 1 animal with mean ± SD. Statistics: one-way ANOVAs with Tukey’s multiple comparison correction, p-values **** < 0.0001, *** < 0.001, ** < 0.01, * < 0.05, no significance > 0.05. RNAi lines: control (JF01355), eIF4B (HMS04503), eIF4H1 (HMS04504). For full genotypes see Additional file 7: Table S1

The effects of co-expressing *eIF4B*, *eIF4H1*, or control (*Luc*) RNAi with LDS-(G4C2)_EXP_ using GMR-GAL4 were analyzed in the eye. Externally, *eIF4B* or *eIF4H1* RNAi caused reduced toxicity compared to the control RNAi, seen by recovered red pigment and ommatidial organization (Fig. 4a). Internally, retinal tissue loss caused by LDS-(G4C2)_EXP_ was also mitigated by depletion of *eIF4B* or *eIF4H1*. Blinded quantification of the total surface area for retina tissue or of tissue depth (at the point of the optic chiasm) revealed that suppression was consistent and significant (Fig. 4b). Suppression was recapitulated with a second set of RNAi lines, supporting that the effects seen are the result of downregulating these target genes (Additional file 1: Figure S4B-C).

To further assess if *eIF4B* and *eIF4H1* could be acting downstream of toxic GR-production, RNAi lines targeting *eIF4B*, *eIF4H1*, or control (*Luc*) were co-expressed with (GR)_36_ in the fly eye using GMR-GAL4. The (GR)_36_ transgene produces a GR dipeptide from a non-G4C2 repeat transcript [53]. In contrast to the effect in LDS-(G4C2)_EXP_ animals, *eIF4B* and *eIF4H1* RNAi increased GR-toxicity in both the external and internal eye (Fig. 4c-d). This argues that these genes do not act on the same pathway in GR animals as in LDS-(G4C2)_EXP_ animals. Depletion of either *eIF4B* or *eIF4H1* on their own did not alter normal eye morphology (Fig. 4e-f).

### Depletion of *eIF4B* or *eIF4H1* reduces GR-production in LDS-(G4C2)_EXP_ animals

**Fig. 5:**
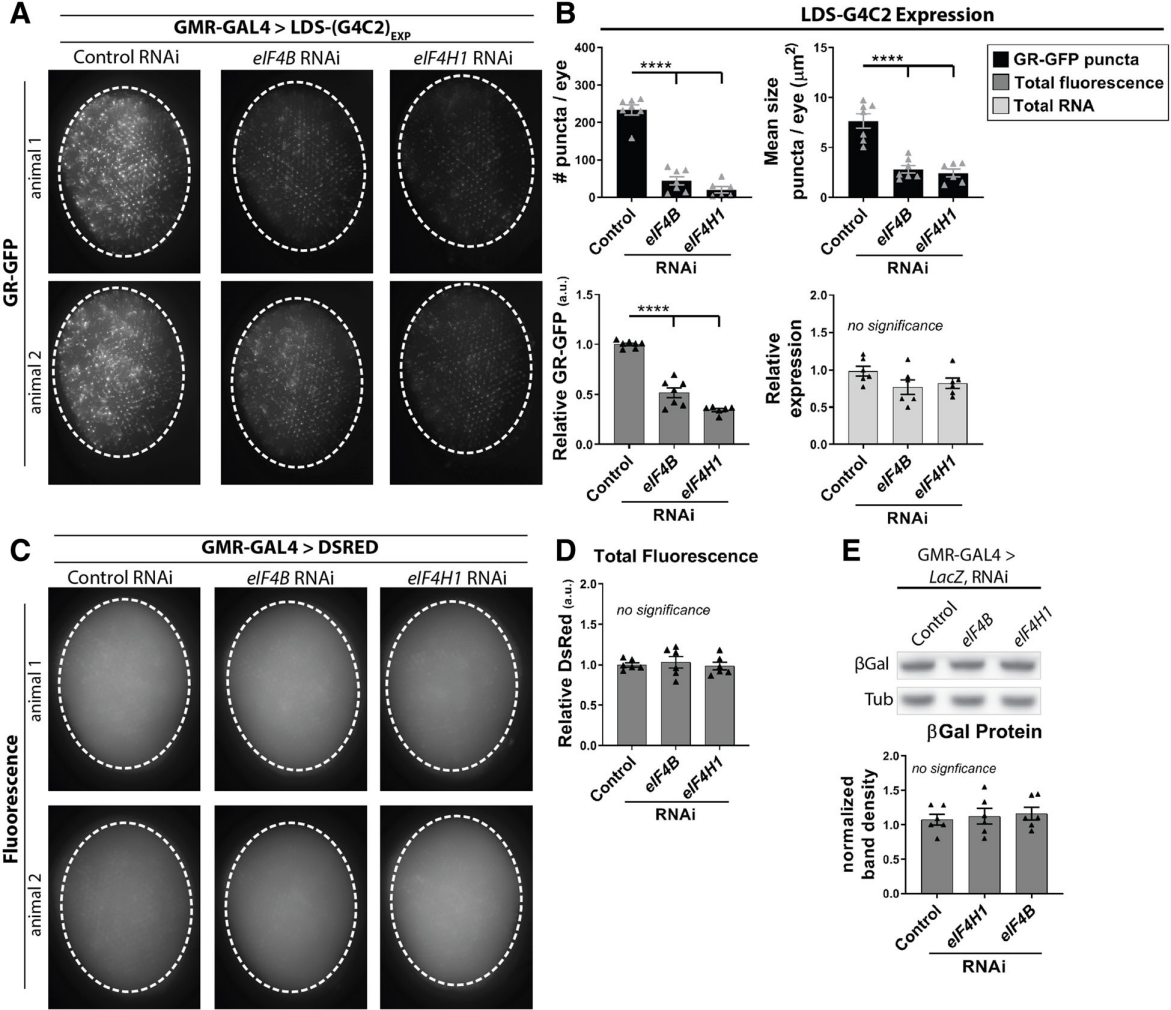
*eIF4B* and *eIF4H1* RNAi selectively reduce GR-GFP levels produced from LDS-(G4C2)_EXP_. **a**. Fluorescence imaging in LDS-(G4C2)_EXP_ expressing flies shows that depletion of *eIF4B* or *eIF4H* by RNAi results in reduced GR-GFP levels (GMR-GAL4). **b**. Blinded quantification of GR-GFP signal in LDS-(G4C2)_EXP_ animals relative to the signal in control RNAi animals. Analysis of GR-GFP puncta number and size are shown in *black*. Total GR-GFP signal is shown in *grey*. *n* = 6–7 animals per genotype. Further, qPCR was used to quantify RNA levels in LDS-(G4C2)_EXP_ flies co-expressing control, *eIF4B*, or *eIF4H1* RNAi, shown in *light grey*. **c**. A control fluorescent protein, DsRed, was similarly expressed in the fly eye with RNAi to control, *eIF4B*, or *eIF4H*. **d**. Blinded quantification of DsRed fluorescence shows no effect by RNAi. n = 6 animals per genotype. **e**. A representative western immunoblot image for β-Galactosidase in *LacZ* flies co-expressing control, *eIF4B*, or *eIF4H1* RNAi. Uncropped westerns (Additional file 1: Figure S4). Blinded quantification of β-Galactosidase western immunoblots normalized to the loading control, Tubulin. For graphs, shown are individual data points from 2 independent assays with mean ± SEM. Statistics: one-way ANOVAs with Tukey’s multiple comparison correction, p-values **** < 0.0001, *** < 0.001, ** < 0.01, * < 0.05, no significance > 0.05. RNAi lines: control (JF01355), *eIF4B* (HMS04503), *eIF4H1* (HMS04504). For full genotypes see Additional file 7: Table S1

As *eIF4B* and *eIF4H1* are canonical translation factors we hypothesized that they modified LDS-(G4C2)_EXP_ toxicity by mediating translation from the G4C2 transcript. To further test if depletion of these factors reduced GR production, *eIF4B*, *eIF4H1*, or control (*Luc*) RNAi were co-expressed with LDS-(G4C2)_EXP_ in the fly eye and fluorescence imaging was performed (Fig. 5a). Blinded quantification of GR-GFP signal in LDS-(G4C2)_EXP_ flies revealed that *eIF4B* depletion caused a 48.3 ± 13% decrease in total GR-GFP fluorescence (Fig. 5b, *grey*). Further, *eIF4H1* depletion caused a 65.5 ± 3.7% decrease in GR-GFP fluorescence levels. As puncta formation was associated with fluorescently tagged GR (see Fig. 1.), additional analyses were performed to define changes in the number of bright GR-GFP puncta and the average size of these puncta (Fig. 5b, *black*). *eIF4B* RNAi reduced the number of puncta from 233 per eye to 44 per eye, an 81% reduction. Additionally, the average size of the puncta per eye was reduced by 63% (7.6μm^2^ to 2.8μm^2^). *eIFH1* RNAi caused a 91% reduction in the number of GR-GFP puncta per eye (233 to 20) and the size of the puncta was reduced from 7.6μm^2^ to 2.4 μm^2^, a 68% reduction. Effects on GR-GFP levels and puncta were also seen using the second set of RNAi lines targeting *eIF4B* and *eIF4H1* (Additional file 1: Figure S3D-E).

While we had already determined that *eIF4B* and *eIF4H1* RNAi did not alter toxicity downstream of GR production (see Fig. 4c-d), we considered whether their depletion could modify LDS-(G4C2)_EXP_ upstream of translation, on the transcriptional level. To assess this, we used qPCR to measure transcript levels of the LDS-(G4C2)_EXP_ transgene in animals co-expressing control RNAi (*Luc*), *eIF4B* or *eIF4H1* RNAi (Fig. 5b, *light grey*). Depletion of *eIF4B* or *eIF4H1* did not alter LDS-(G4C2)_EXP_ RNA levels, further supporting that they act on the translational level.

To test the specificity of *eIF4B* and *eIF4H1* to translation of a G4C2 transcript, we first confirmed specificity of the effect of *eIF4B* and *eIF4H1* RNAi, by assessing whether their depletion had an effect on eye fluorescence of a control DsRed transgene in the fly optic system (GMR-GAL4) (Fig. 5c). Blinded quantification supported that total fluorescence was unchanged, arguing that the effect in LDS-(G4C2)_EXP_ animals was specific to the GR-tagged fluorescent protein (Fig. 5d). We further used western immunoblots to analyze protein levels produced from a control (*LacZ*) transgene in animals co-expressing *eIF4B*, *eIF4H1*, or control (*Luc*) RNAi (Fig. 5e). Transgenes were expressed using GMR-GAL4 and protein was extracted from whole heads. Consistent with fluorescence data using DsRed, no significant difference in the amount of β-galactosidase protein translated from the *LacZ* transcript was seen. The second set of RNAi lines targeting *eIF4B* and *eIF4H1* also did not alter protein expression from a control (*LacZ)* gene (Additional file 1: Figure S3F). These data are consistent with previous reports that these factors are not essential for general translation [2, 11, 15, 33].

Overall, these data support that *eIF4B* or *eIF4H1* modify LDS-(G4C2)_EXP_ toxicity by mediating toxic GR production. Importantly, translation from the G4C2 transcript is particularly sensitive to their depletion as expression from control transcripts were unaltered under similar conditions.

### *EIF4H* is downregulated in ALS/FTD cases harboring a G4C2 expansion in *C9orf72*

Data in the fly supported that *eIF4B* and *eIF4H1* were modifiers of LDS-(G4C2)_EXP_ that could alter the amount of GR produced from the repeat-containing transcript. To further investigate these translation factors in disease, we considered that the expression of the human orthologues to these factors, *eIF4B* and *eIF4H*, could be dysregulated if they played a critical role in G4C2-associated expression.

**Fig. 6:**
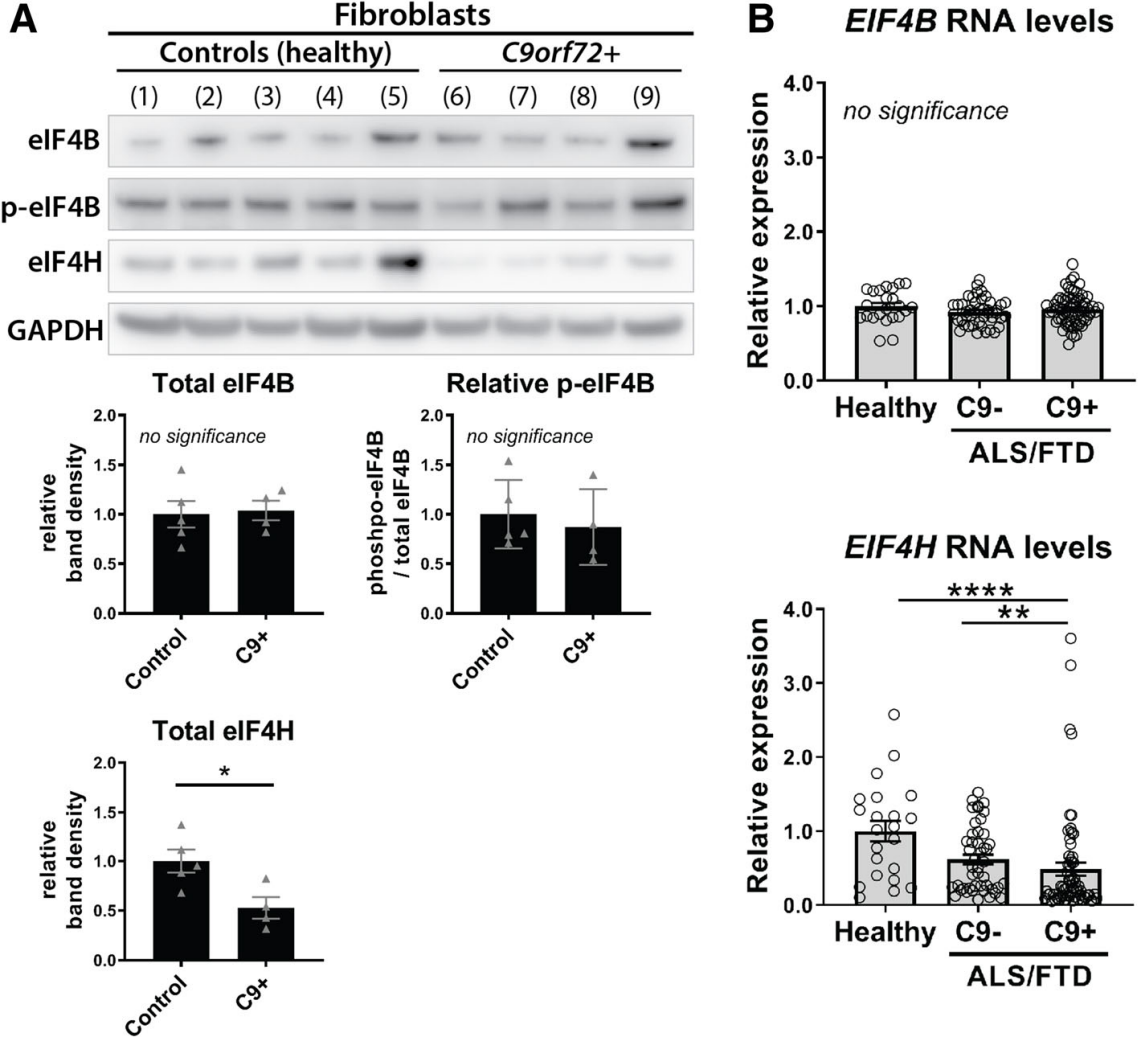
*EIF4H* is downregulated in C9+ ALS/FTD. **a**. Western immunoblots were used to define changes in eIF4B or eIF4H protein levels from 5 independent control or 4 independent C9+ derived fibroblast cell lines. Data are relative to controls. Quantification of total protein was done after normalizing to loading, using GAPDH. Phospho-eIF4B quantification was further normalized to total eIF4B. Statistics: unpaired student t-tests. Shown: each data point represents 1 cell line with mean ± SEM; the mean data from 2 independent protein preparations is shown per line. **b**. RNA levels of *EIF4B* or *EIF4H* were assessed by qPCR in human cerebellar tissue from healthy individuals or ALS/FTD patients with (C9+) or without (C9-) the G4C2 expansion in *C9orf72*. *n* = 22 (healthy), 46 (C9- ALS/FTD), 66 (C9+ ALS/FTD). Statistics: one-way ANOVAs with Dunn’s multiple comparison correction. Shown: individual data points representing 1 individual with mean ± SEM. Cell line details: Additional file 3: Table S3. Patient details: Additional file 4: Table S4. *p*-values **** < 0.0001, *** < 0.001, ** < 0.01, * < 0.05, no significance > 0.05

eIF4B and eIF4H protein levels were assessed by western immunoblot in primary fibroblast cell lines (Fig. 6.A; lines described in Additional file 3: Table S3). The mean expression from four independent C9 + patient derived lines was compared to the mean expression from five independent lines derived from healthy individuals. Interestingly, eIF4B total levels were unchanged while eIF4H levels were reduced by 47.5%. As eIF4B is inhibited by phosphorylation at Ser422 [64, 69], we further examined levels of phospho-eIF4B to determine whether this factor was dysregulated by protein modification. No obvious changes were observed in phospho-eIF4B levels visually or relative to total eIF4B in C9+ versus healthy cells.

Data from patient-derived cells supported that eIF4H is dysregulated in C9+ situations. To further assess this finding in patients, total RNA was extracted from post-mortem, cerebellar tissue from 112 ALS/FTD individuals or 22 healthy individuals and the expression from *eIF4B* or *eIF4H* were defined by qPCR (Fig. 6b; individuals described in Additional file 4: Table S4). The ALS/FTD cohort were further broken down based on the presence or absence of the G4C2-repeat expansion in *C9orf72* into 46 C9- ALS/FTD and 66 C9+ ALS/FTD cases. Consistent with protein data from fibroblast lines, *eIF4B* expression was unaltered in disease. Importantly, *eIF4H* was significantly downregulated by 71.2% in C9+ ALS/FTD compared to healthy controls and 54.4% compared to C9- ALS/FTD cases.

These data indicate a significant decrease in *eIF4H* expression in response to the presence of expanded G4C2 in ALS/FTD.

## Discussion

Mechanisms underlying repeat-associated non-AUG (RAN-) translation remain unclear despite evidence that this form of translation occurs in disease [37, 96]. To help define potential RAN-translation factors, we developed a gain-of-function fly model for *C9orf72-*associated ALS/FTD that expressed an expanded GGGGCC hexanucleotide repeat (termed G4C2) downstream of the sequence normally found upstream of the repeat in patients (114 bp of intronic DNA found 5′-prime of the repeat in intron 1 of *C9orf72* in ALS/FTD); the transgene expressed also contained a GFP tag downstream of the repeat in the GR reading frame (see Fig. 1). Using this model, we screened 48 of 56 canonical translation factors in flies [50] to define those that could impact expression of the GR dipeptide (see Fig. 2 and Additional file 2: Table S2). 11 candidate RAN-translation factors were defined (see Table 1). When depleted, these factors reduced GR-GFP levels, reduced G4C2-induced toxicity, and did not reduce toxicity associated with a non-G4C2 transcript generated toxic GR protein. Further investigations into two of these, *eIF4B* and *eIF4H1* (fly orthologue to *eIF4H*), revealed that their depletion reduced G4C2-induced toxicity and GR-GFP levels, but did not alter G4C2 RNA levels (see Fig. 5 and Additional file 1: Figure S3). Investigations into *eIF4B* and *eIF4H* expression in patient-derived cells and in post-mortem tissue revealed that *eIF4H* is significantly downregulated in ALS/FTD patients harboring the G4C2 expansion (C9+ ALS/FTD) (see Fig. 6). This effect was not seen in ALS/FTD patients lacking the G4C2 mutation (C9- ALS/FTD), arguing that it is a response to the presence of the repeat. These data highlight *eIF4B* and *eIF4H* as novel factors mediating disease-associated pathways.

**Fig. 7:**
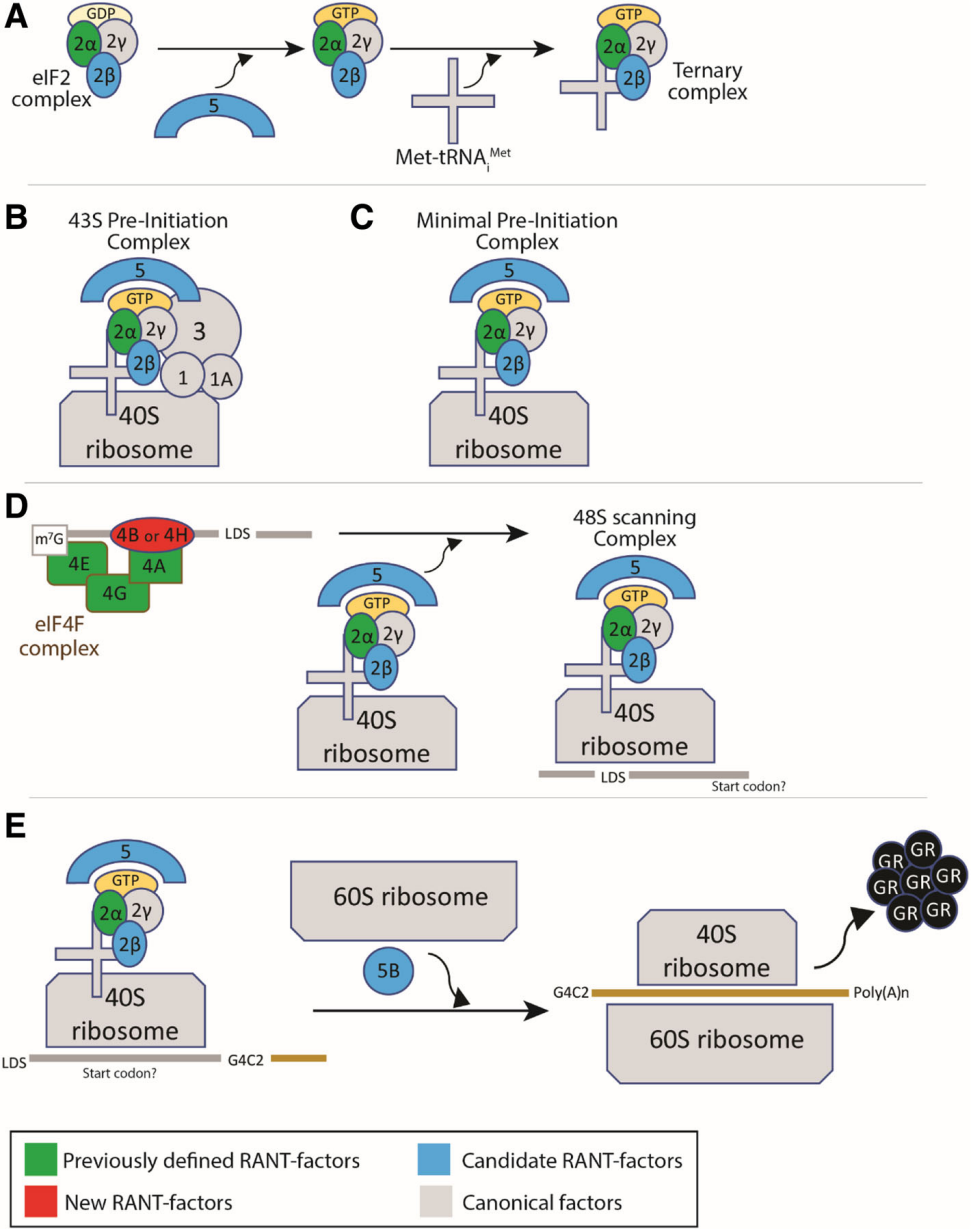
Model comparing potential G4C2 RAN-translation mechanisms and canonical translation. **a**. Ternary complex formation requires eIF5-mediated exchange of GDP to GTP on eIF2 complex (includes eIFs 2α, 2β, 2γ). eIF2α is highly regulated during stress and is reported to mediate G4C2 translation [12, 27]. eIF2β and eIF5 were identified as modifiers in this study. **b**. In normal translation, the formation of the 43S pre-initiation complex (PIC) involves the joining of a number of factors, including Ternary complex (described in a) and eIFs 1, 1A, 3, and 5. **c**. A minimal PIC complex may potentially mediate RAN-translation [1, 42, 76, 86]. **d**. mRNA transcripts are recognized by the eIF4F complex, includes eIFs 4E, 4G, 4A. All of these have been defined as G4C2 translation factors arguing that G4C2 RAN-translation is cap-dependent [12, 84]. eIF4E recognizes the 5-prime m^7^G cap on mRNAs [10, 77, 78]; notably, 4 of 6 eIF4E components were identified in our screen. eIF4A is recruited by eIF4E to mRNA transcripts (via the scaffold protein eIF4G). mRNA is then unwound by eIF4A, an activity that is significantly promoted by eIF4B or eIF4H, identified herein [24, 68, 70, 82, 91]. This action allows for the formation of the 48S scanning complex. **e**. In canonical translation, the 48S scanning complex moves down a transcript until identifying an AUG start codon. A CUG codon in the LDS sequence upstream of G4C2 may function as a start codon in the GA-reading frame [27, 84]. Frame-shifting could allow for translation of the GR and GP from this codon. Candidate RAN translation factors eIF5B, and potentially eIF5, mediate ribosome scanning, start codon recognition, and translation activation [10, 45, 61]. *We note that mechanisms underlying RAN-translation are still relatively unknown. This model is based on current literature and canonical functions of translation factors*

To our knowledge, this is the first in vivo investigation into canonical translation factors that impact dipeptide production in a *C9orf72*-associated disease model. The simplest hypothesis is that these factors impact the process of RAN translation from the G4C2 repeat-containing transcript. Interestingly, our data suggest that production of the GR dipeptide requires specific factors as only 11 of 48 canonical translation factors screened altered GR-GFP levels in G4C2-expressing animals. Further, investigations into mammalian systems and in disease-relevant tissue using DPR-specific antibodies will determine if *eIF4B* or *eIF4H* depletion disrupts expression of multiple DPR and confirm effects on GR production [98]. Although these factors could function to alter GR-production through alternative means versus RAN-translation (i.e. stability of the protein, altered expression of more direct RAN-translation factors), it is compelling that the factors identified converge at key regulatory steps of translation initiation and that the majority of these factors function together or with previously suggested RAN-translation factors (see Fig. 7). We ruled out factors that similarly altered toxicity caused by the GR dipeptide, supporting that they act on pathways not associated with toxicity of the GR protein. We also ruled out mechanisms underlying G4C2 transcription and RNA stability for *eIF4B* and *eIF4H*, as G4C2 transcript levels are unaltered by their depletion (see Fig. 5b). Although our investigations here focused on *eIF4B* and *eIF4H*, we note that we defined a number of other intriguing factors that act on either on G4C2- and/or GR-associated toxicity (see Table 1).

The fly model developed herein expresses the LDS-G4C2 transcript as an mRNA, containing a 5′-prime m^7^G cap and polyadenylated (poly(A)-) tail. In disease, the G4C2-RNA could exist in multiple forms [93], including: improperly spliced *C9orf72*-mRNA transcripts retaining the repeat [58], properly spliced intronic sequence kept stable by the repeat [30, 90], altered transcription initiation products [71], or aborted *C9orf72*-transcript products [29]. Interestingly, fly models that express expanded G4C2 within properly spliced introns do not show dipeptide expression nor toxicity, despite the formation of RNA foci [88]. Further, improperly spliced *C9orf72*-transcripts can be shuttled from the nucleus to the cytoplasm where they undergo RAN-translation [32]. Overall, these data support that G4C2 repeats retained in capped and polyadenylated *C9orf72*-mRNA are able to produce toxic dipeptides and are relevant to patients. Whether or not G4C2-associated RAN translation is dependent on the presence of this 5′-prime cap (and poly(A)-tail) is still debated [12, 27, 84].

Our investigations draw parallels between the canonical functions of translation factors [50] and RAN-translation (see Model, Fig. 7). However, there are multiple types of translation that may be pertinent to disease, including cap-independent mechanisms, such as IRES translation [28, 37, 76, 77]. Of the 11 candidate RAN-translation factors we identified, 4 have been reported to function in cap-independent translation: eIF4B [38, 75], eIF4H [91], eIF5B [23], eIF2β [44]. Interestingly, eIF4B or eIF4H significantly strengthen eIF4A-mediated unwinding of longer, more complex 5′ UTRs in transcripts [70, 74, 82, 91]. This activity is thought to mediate scanning of the 5′ UTR for translation start sites [79] and recruitment of ribosomal subunits [75, 92]. Overall, these data suggest a model for G4C2-associated RAN-translation where eIF4B/eIF4H and eIF4A mediate dipeptide production during this scanning process [28, 84]. Further, frame-shifting during scanning could result in the production of all three sense-strand associated dipeptides (GA, GR, GP) from a near-cognate alternative start codon, CUG, found upstream of the repeat in the GA-reading frame [84].

In addition to their involvement in non-canonical translation and in stimulating eIF4A (previously reported as a RAN-translation factor [27, 84]), we chose to focus on eIF4B and eIF4H as they are RNA-binding proteins (RBPs) containing homologous RNA recognition motifs (RRMs) [66]. Screens for RBPs that interact with G4C2-RNA identified both eIF4B and eIF4H, supporting our data that they function in translation from G4C2 transcripts [14, 29, 72]. Both eIF4B and eIF4H can independently stimulate the helicase activity of eIF4A during translation [24, 31, 59, 68, 70, 74, 82, 91] while key differences between them are noted. Structural data supports that eIF4H is constitutively active while eIF4B contains a regulatory carboxyl domain containing multiple phosphorylation sites [52, 64]. This may explain why *eIF4H* is downregulated in C9+ ALS/FTD but not *eIF4B* (see Fig. 6), as eIF4B can be regulated by de/phosphorylation. However, no significant changes were observed for phospho-eIF4B at Ser422, an inhibitory modification [69, 77], in four C9+ derived cell lines versus five control cell lines. Extended analyses are needed to increase the sample size and to test for eIF4B phosphorylation at other marks [9, 89]. Overall, we hypothesize that *eIF4H* is downregulated as the result of compensatory mechanisms: cells may actively downregulate *eIF4H* to reduce expression of toxic GR dipeptide. Alternatively, as eIF4H had previously been reported to bind G4C2-RNA [14, 29, 72], the reduced *eIF4H* RNA levels could be the result of a more complex feedback loop: as eIF4H protein is sequestered by G4C2 RNA foci, cells may respond by downregulating *eIF4H* transcription as they may sense that there is plenty of this translation factor present. However, the eIF4H that is present may not be functioning normally. Localization studies in patient tissue are needed to determine if eIF4H is indeed sequestered into G4C2-foci. In either scenario, data supports that eIF4H plays an important role in C9+ disease while further investigations would help define its role in disease progression.

Studies in multiple model systems support that *eIF4B* and *eIF4H* loss does not inhibit global translation, including yeast [2, 15], flies [33], and mice [11]. Interestingly, yeast and flies (see Fig. 3b) with downregulated *eIF4B* or *eIF4H* are viable [2, 15, 33] and *eIF4H*^*+/−*^ mice do not have notable deficits [11]; although *eIF4B* and *eIF4H* have been suggested to be important for brain development [9, 11, 20, 67]. The downstream consequences of *eIF4H* downregulation in C9+ ALS/FTD may be broader than simply altering RAN-translation as depletion of *eIF4B* and *eIF4H* in cultured cells has been shown to induce stress granule formation [54]. Interestingly, since *eIF4H* expression is reduced in C9+ ALS/FTD and C9+ derived cells (see Fig. 6), this raises a potential connection to mechanisms underlying TDP-43 pathology/toxicity [16, 21]. Further, our data in GR-expressing flies argues that the depletion of these factors downstream of GR-production can feed into pathways disrupted by this toxic dipeptide (see Fig. 4c-d) [35, 83, 85, 94].

In conclusion, in an unbiased, targeted screen we identified *eIF4B* and *eIF4H* as canonical translation factors that, when depleted in flies, disrupted toxicity caused by the expression of expanded G4C2 RNA. Interestingly, *eIF4H* was downregulated in C9+ ALS/FTD patients, indicating a distinct role in *C9orf72*-associated disease. These factors may represent unique G4C2 modifiers that couple RAN-translation to dysregulation of RNA metabolism in disease [16, 34, 95].

## Methods and materials

### Patient samples and clinical, genetic and pathological assessments

Participant information is summarized in Additional file 4: Table S4. Protocols were approved by the Mayo Clinic Institutional Review Board and Ethics Committee. All participants (or authorized family members) were provided written informed consent before information gathering, autopsies and postmortem analyses. Trained neurologists diagnosed patients with ALS and/or FTD after reviewing neurological and pathological information. The presence or absence of an expanded G4C2 within intron 1 of *C9orf72* was done using a previously established protocol for repeat-primed polymerase chain reaction [17].

### *Drosophila* work

Stocks were maintained on standard cornmeal-molasses medium. Fly lines used are detailed in Additional file 2: Table S2 and Additional file 5: Table S5. Fly lines obtained from Bloomington *Drosophila* Stock Center (BDSC) and Vienna *Drosophila* Resource Center (VDRC) are noted.

### Fly RNAi efficacy

All control and RNAi lines are defined in Additional file 5: Table S5. RNAi efficacy was determined using Da-GAL4 (adults or larvae) as previously described [26, 51].

### Characterization of LDS-(G4C2)n fly models

Transgenes were inserted into pUAST vectors and randomly inserted into *w*^*1118*^ fly genomes. The LDS-G4C2 model has a 5′ leader sequence (LDS) inserted immediately upstream of the G4C2 repeats, 114 bp of sequence upstream of the repeat in intron 1 of *C9orf72* in patients, and a 3′ GFP tag in the GR reading frame. **Repeat-length determination:** Genomic DNA was extracted from individual fly lines and the transgenes present were amplified by PCR using primers designed to flank the repeat (Additional file 6: Table S6). Amplification was done using a KAPA HiFi HotStart kit (Kappa #KK2501) and PCR product sizes were quantified using agarose gels and a Bioanalyzer, previously described [26]. Control *w*^*1118*^ animals were included in experiments and showed no signal. **RNA expression:** Transgenes were expressed as previously described using HS-Gal4 [26] and RNA levels were assessed by qPCR, using primers designed to amplify the GFP tag (Additional file 6: Table S6). Control *w*^*1118*^ animals were included in experiments and showed no signal.

### LOF external fly eye screen

Publicly available RNAi [57, 60] or mutant [6, 7, 80, 81] loss-of-function (LOF) fly lines targeting canonical translation factors were obtained from the Bloomington *Drosophila* Stock Center (BDSC). Additional UAS-RNAi lines targeting *eIF4B* and *eIF4H1* were obtained from Vienna *Drosophila* Resource Center (VDRC) [18].

#### External eye imaging

LOF males were crossed to recombinant females: UAS-LDS-(G4C2)_EXP_, GMR-GAL4 (III) (26 °C). Multiple *w*^*−*^ and *w*^*+*^ controls were setup with every experiment to assess any natural variability, including a UAS-Luc RNAi (BDSC # 31603) and *w*^*1118*^; UAS-DSRED. External eyes for 1-2d progeny were imaged on a Leica Z16 APO microscope as described [26]. Any changes to the ommatidia organization, eye size, pigmentation, and ability to eclose from pupae were noted. Resulting phenotype was categorized into one of six groups: suppressors, mild suppressors, no effect, mild enhancers, enhancers, and lethal enhancers (Additional file 1: Figure S2).

#### External eye fluorescence imaging

LOF males were crossed to recombinant females: UAS-LDS-(G4C2)_EXP_, GMR-GAL4 (III) (26 °C) and 1-2d progeny were imaged on a Leica DM6000B and quantified as previously described [26]. *w*^*−*^ or *w*^*+*^ controls were used for accurate comparisons depending on the background of the LOF lines and the final genotypes of animals. Any changes to the GR-GFP levels using LUT Z-stacked images were noted. Resulting phenotype was categorized into one of six groups: suppressors, mild suppressors, no effect, mild enhancers, enhancers, and lethal enhancers (Additional file 1: Figure S2). Researchers were blinded to the LOF targets during screening. Modifiers of LDS-(G4C2)_EXP_ toxicity and/or GR-GFP levels were further assessed in GMR-GAL4 > UAS-(GR)36 animals. Modifier crosses were repeated 3+ independent times to confirm reproducibility of results.

#### Quality control experiments

Defining unspecific LOF lines were performed as previously described [26].

### Fibroblast cells

Cultured using standard protocols in DMEM complete media: 15% FBS (Sigma), 1x MEM Amino Acids (ThermoSci # 11130051), 1% pen/strep, DMEM (high glucose, plus sodium pyruvate). Cells were maintained in a 5% CO_2_ incubator at 37 °C.

### Western immunoblots (WB)

**Fly tissue:** Triplicate samples of 5–10 heads per genotype were homogenized using disposable pellet/pestles tissue grinders (Kimble Chase #749520–0000) and motor (Kimble Chase #749540–0000). For βgal: heads were directly homogenized into 1X NuPAGE LDS sample buffer. For GR/GFP: heads were homogenized into RIPA buffer (50 mM Tris-HCL (pH 7.5), 150 mM NaCl, 1% NP-40, 50 mM NaF, 0.5% DOC), plus protease inhibitors (Sigma # 05892970001), 1 mM PMSF, and 1 mM DTT. **Fibroblast cells**: cells were lysed in RIPA buffer plus protease inhibitors, 1 mM PMSF, and 1 mM DTT, and phosphatase inhibitors (Sigma # 04906845001) for 30 min at 4 °C. **All RIPA lysates**: quantified by Bradford; 20 μg of protein was run per lane. **All WBs**: run using a standard protocol with Invitrogen’s XCell SureLock blot system, 4–12% Bis-Tris NuPAGE gels and a wet transfer with PVDF membrane, except βgal which was transferred with an iBlot dry transfer system (program 2, 8 min) and nitrocellulose membrane. **Antibodies**: anti-βgalactosidase (Promega #Z3781, 1:2000), anti-αTubulin (DSHB #AA4.3, 1:2000), anti-GFP^JL8^ (Takara #632380, 1:10,000), anti-GR (gift from V. M-Y. Lee #2316, 1:1000). ***H. sapiens*** **antibodies**: anti-eIF4B (Cell Signaling #3592, 1:1000), anti-eIF4H (Cell Signaling #3469,1:1000), anti-phospho-eIF4B (Cell Signaling #3591,1:1000), anti-GAPDH (Sigma #G8795, 1:5000). **Secondary antibodies**: Mouse-HRP (Jackson Immunoresearch Labs #115–035-146, 1:5000), Rabbit-HRP (Jackson Immunoresearch Labs #111–035-144, 1:5000) [8]. Blots were analyzed using Amersham ECL Prime Detection Reagent and imaged on an Amersham Imager 600.

### Quantitative real-time PCR (qPCR)

All primers are defined in Additional file 6: Table S6. For both fly and human qPCRs, protocols are previously described with the following changes [26]. **Flies:** For Da-GAL4 assays, triplicate samples of 5 whole animals were processed per condition. For GMR-GAL4 assays, triplicate samples of 20 fly heads (1-2d) were processed per condition. **Humans:** Total RNA was extracted from frozen postmortem tissue from the cerebellum using the RNAeasy Plus Mini Kit (QIAGEN), previously described [62]. RNA integrity (RIN) was verified on an Agilent 2100 bioanalyzer. RIN values ranged from 6.7 to 10, with most of the samples falling between 9.1 and 9.8.

### Statistical analysis and data availability

GraphPad Prism 8.00 software was used to develop all graphs and for all statistical analyses. *P*-values < 0.05 were considered significant. All relevant data are included within the manuscript and supplementary data. Additional inquiries can be directed to the corresponding author, including reagent requests. No statistical methods were used to predetermine sample sizes and data distributions were assumed to be normal, similar to previous work [8, 19, 26, 39, 40, 51, 53, 62, 63]. **Fly and fibroblast data:** A two-tailed unpaired student t-test or one-way ANOVA with Tukey’s multiple comparisons test was performed when appropriate. Researchers were blinded to the genotype of all samples to maintain unbiased scoring. **Human qPCRs:** Nonparametric, one-way ANOVAs with Dunn’s multiple comparisons test were performed as data distribution was not normal.

## Additional files

Additional file 1: **Figure** **S1.** Extended LDS-G4C2 fly line characterization. **Figure S2.** Extended translation factor screen data. **Figure S3.** *eIF4B* and *eIF4H1* RNAi-2 data. **Figure S4.** Full western immunoblot images. (DOCX 1150 kb)

Additional file 2: **Table S2.** Translation Factor screen details. (PDF 157 kb)

Additional file 3: **Table S3.** Fibroblast lines. (PDF 56 kb)

Additional file 4: **Table S4.** Patient samples. (PDF 34 kb)

Additional file 5: **Table S5.** Fly lines used. (PDF 80 kb)

Additional file 6: **Table S6.** primers. (PDF 74 kb)

Additional file 7: **Table S1.** Full genotypes in figures. (PDF 121 kb)

## Abbreviations

ALS: Amyotrophic Lateral Sclerosis
C9- ALS/FTD: ALS/FTD cases lacking the G4C2 expansion in *C9orf72*
C9+ ALS/FTD: ALS/FTD cases harboring the G4C2 expansion in *C9orf72*
DPR: Dipeptide repeats
FTD: Frontotemporal Dementia
G4C2: Expanded (GGGGCC)_30+_ mutation found within *C9orf72*
GFP: Green fluorescent protein
qPCR: quantitative reverse transcribed polymerase chain reaction
RAN-translation: Repeat Associated Non-AUG translation; also RANT
RBP: RNA binding protein
RRM: RNA recognition motif
WT: Wild-type
