# Supplementary material for: eIF4B and eIF4H mediate GR production from expanded G4C2 in a Drosophila model for C9orf72-associated ALS
Source: Acta Neuropathol Commun. 2019 Apr 25;7:62. doi: 10.1186/s40478-019-0711-9 (PMC6485101; doi:10.1186/s40478-019-0711-9)
Supplement: Supplementary file 1 — Figure S1. Extended LDS-G4C2 fly line characterization. Figure S2. Extended translation factor screen data. Figure S3. eIF4B and eIF4H1 RNAi-2 data. Figure S4. Full western immunoblot images. (DOCX 1150 kb) [file 40478_2019_711_MOESM1_ESM.docx]

***eIF4B and eIF4H mediate GR production from expanded G4C2 in a Drosophila model for C9orf72-associated ALS.***

Lindsey D. Goodman^1^, Mercedes Prudencio^3^, Ananth R. Srinivasan^2^, Olivia M. Rifai^2^, Virginia M-Y. Lee^4^, Leonard Petrucelli^3^, Nancy M. Bonini^1,2^.

# **ADDITIONAL FIGURES**

## **Figure S1**

**
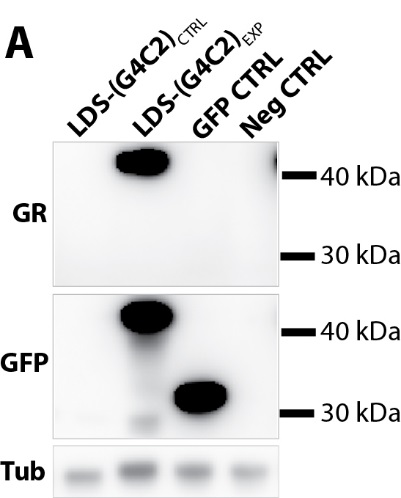
**

**Figure S1: Extended LDS-G4C2 fly line characterization.**

Western immunoblots were used to confirm GR dipeptide is produced and successfully tagged with GFP in expanded G4C2 flies **(Fig. 1D)**. Blots were overexposed to see if GR/GFP dipeptide could be detected in control G4C2 flies. Shown is a representative image of the original blot, imaged at > 4X the original exposure. > 10X the original exposure also showed no GR/GFP dipeptide in control G4C2 flies (data not shown). Note that the faint band at a molecular weight below GFP alone in the LDS-(G4C2)_EXP_ lane is a non-specific band that is found in multiple lanes of the complete western immunoblot (see **Supplementary Fig. 4** for Uncropped westerns)**.**

## **Figure S2**

**
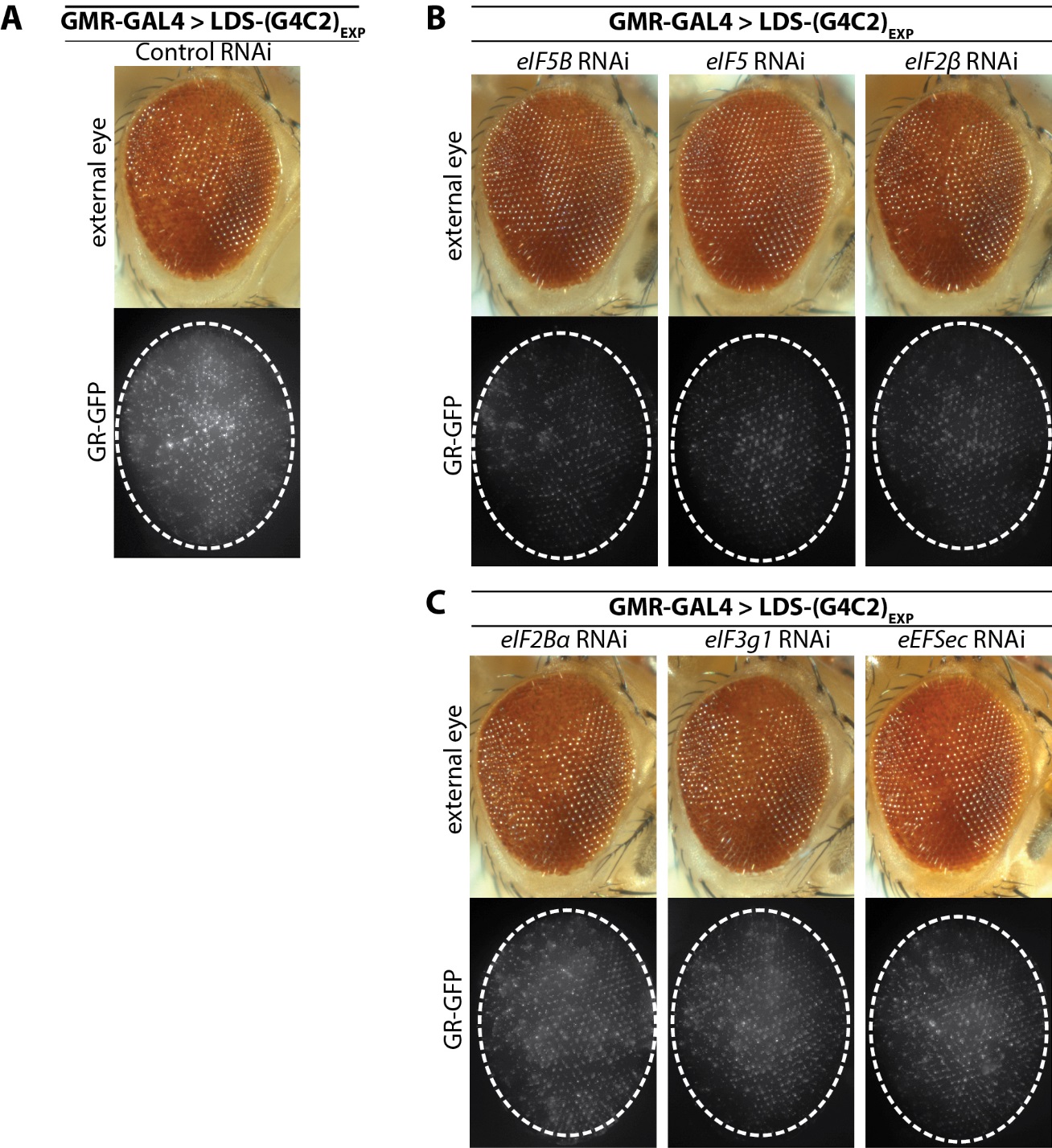
**

**Figure S2: Extended translation factor screen data.**

**A.** External eye and GR-GFP imaging in LDS-(G4C2)_EXP_ animals co-expressing control (*Luc*) RNAi. **B.** Additional examples of candidate RAN-translation factors, showing suppression of toxicity in the external eye and reduced GR-GFP levels. Note, *eIF2β* RNAi only mildly altered toxicity in the external eye. **C.** Examples of RNAi targeting translation factors that did not significantly alter LDS-(G4C2)_EXP_ toxicity nor GR-GFP levels. RNAi lines: control (JF01355), *eIF5B* (GL01593), *eIF5* (HMS00159), *eIF2β* (HMC02396), *eIF2Bα* (HMC03768), *eIF3g1* (GLC01430), *eEFSec* (GL01178). For full genotypes see **Supplementary Table 1**. Shown: representative images while all RNAi were tested 2+ times for effects on LDS-(G4C2)_EXP_ toxicity and GR-GFP levels.

## **Figure S3**

**
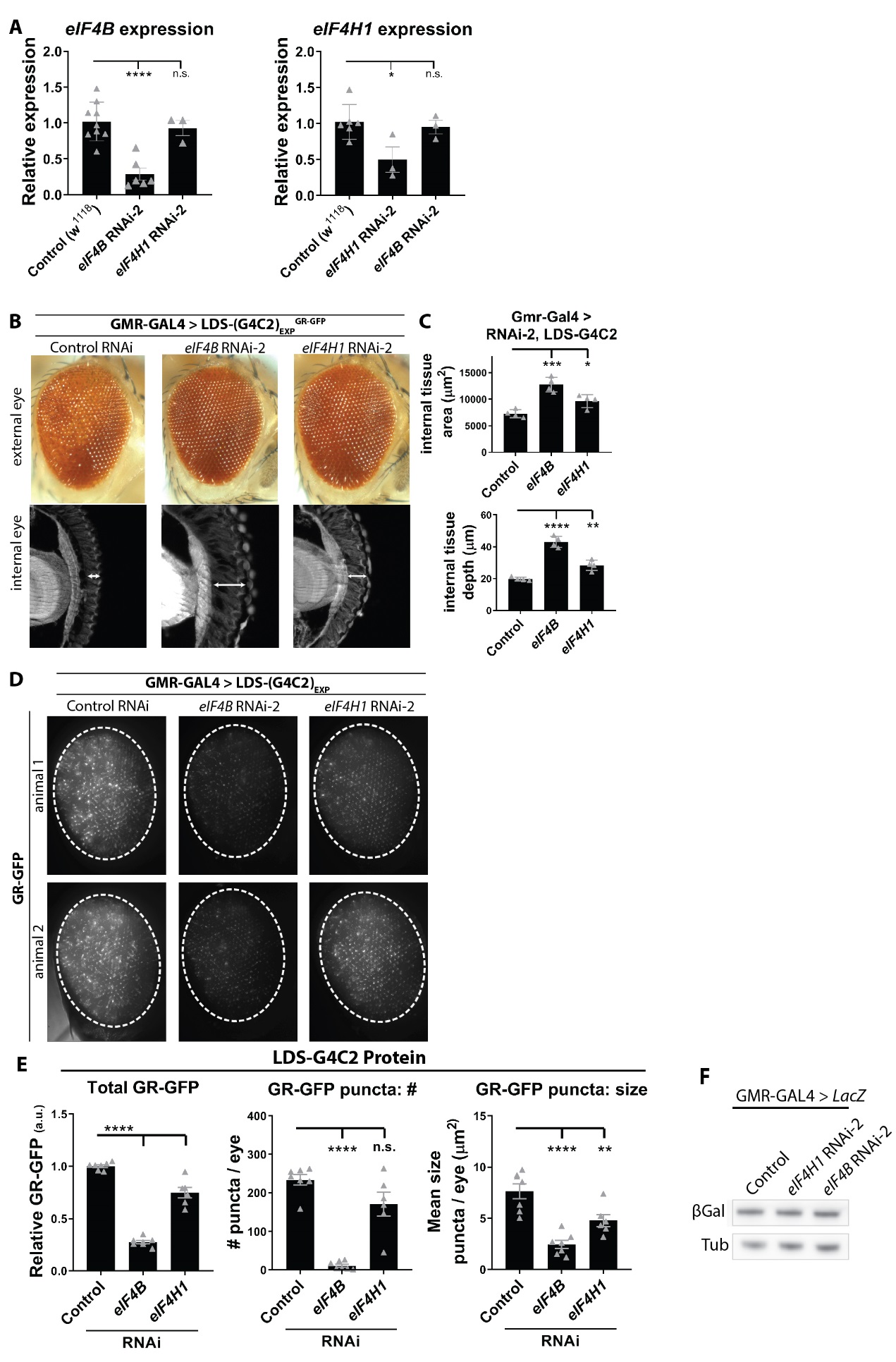
**

**Figure S3: *eIF4B* and *eIF4H1* RNAi-2 data.**

*eIF4B* and *eIF4H1* downregulation in LDS-(G4C2)_EXP_ flies using a second set of RNAi lines show similar results. **A.** RNA levels produced from *eIF4B* or *eIF4H1* were assessed by qPCR in flies ubiquitously expressing RNAi-2 (by Daughterless-GAL4 driver). Shown: individual data points with mean±SD. **B.** Using GMR-GAL4, RNAi-2-mediated depletion of *eIF4B* or *eIF4H1* in LDS-(G4C2)_EXP_ expressing flies results in reduced toxicity in both the external and internal eye: seen externally by recovered pigment and ommatidial structure, seen internally by recovered retinal tissue integrity. **C.** Blinded quantification of internal retina tissue was done by measuring the total surface area of tissue present and by measuring the depth of the tissue at the position of the optic chiasm. n=4 animals per genotype. Shown: individual data points each representing 1 animal, with mean±SD. **D.** Fluorescence imaging in LDS-(G4C2)_EXP_ expressing flies co-expressing *eIF4B* or *eIF4H* RNAi-2 results in reduced GR-GFP levels (GMR-GAL4). **E.** Blinded quantification of GR-GFP signal in LDS-(G4C2)_EXP_ animals relative to signal in control RNAi animals. Note that *eIF4H1* RNAi-2 did not cause a significant reduction in GR-GFP puncta number, likely due to this RNAi only causing a 50% downregulation of *eIF4H1*; it did reduce puncta size and total GR-GFP fluorescence. n=6-7 animals per genotype. Shown: individual data points each representing 1 animal from two independent experiments with mean±SEM. **F.** A representative western immunoblot image for β-Galactosidase in *LacZ* flies co-expressing a control transgene, *eIF4B* RNAi-2, or *eIF4H1* RNAi-2. Uncropped westerns (**Sup. Fig. 4).** Statistics: one-way ANOVAs with Tukey’s multiple comparison correction, p-values ****<0.0001, ***<0.001, **<0.01, *<0.05, no significance >0.05. RNAi lines: control (JF01355), *eIF4B* RNAi-2 (VSH330010), *eIF4H1* RNAi-2 (KK108805). For full genotypes see **Supplementary Table 1**.

## **Figure S4**


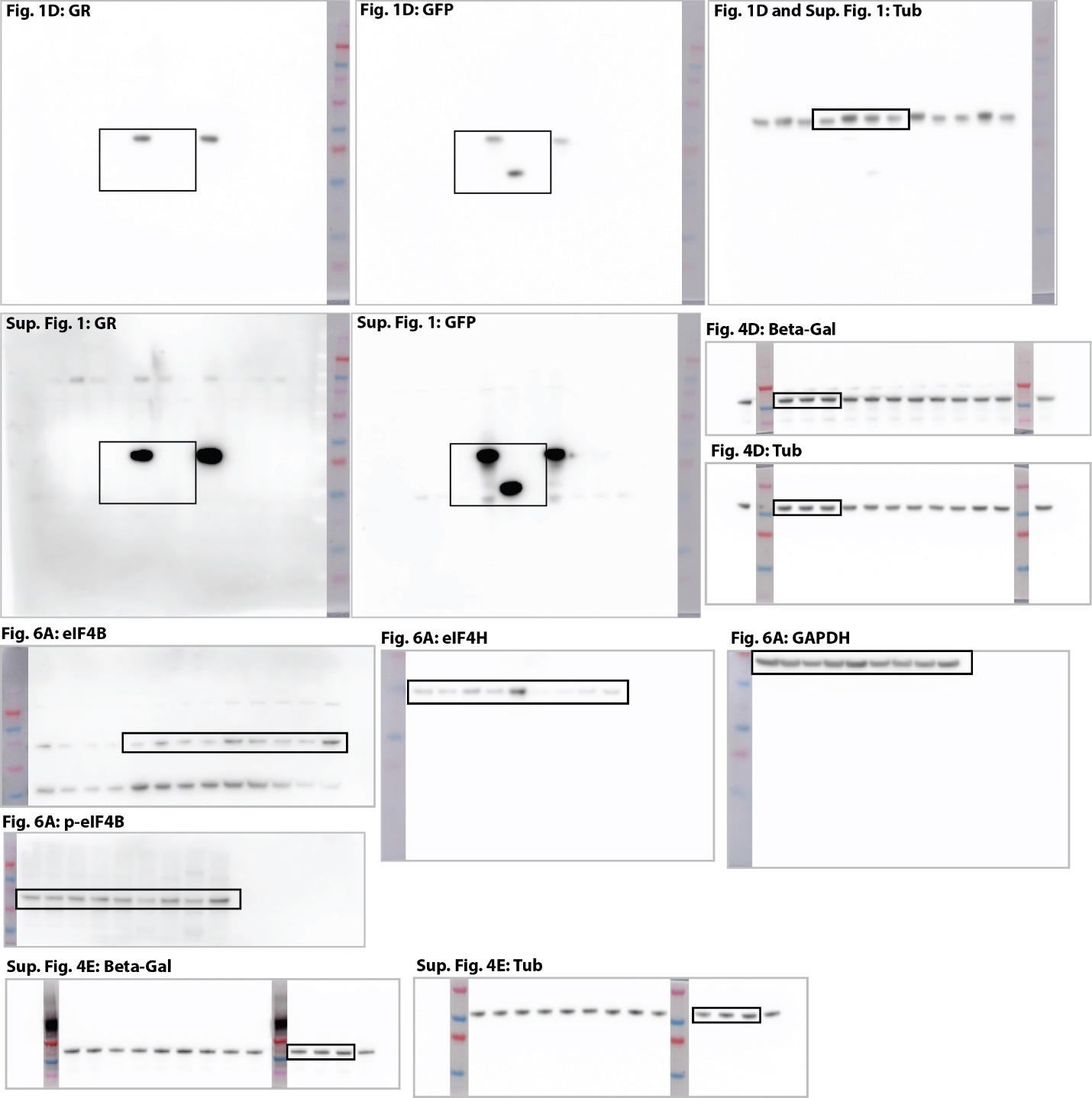


**Figure S4:** full Western immunoblot images for Fig. 1D, Sup. Fig. 1, Fig. 4D, Fig. 6A and Sup. Fig. 4E, including ladder, Novex sharp Protein Standard (Invitrogen #LC5800)
