# Supplementary material for: eIF4B and eIF4H mediate GR production from expanded G4C2 in a Drosophila model for C9orf72-associated ALS
Source: Acta Neuropathol Commun. 2019 Apr 25;7:62. doi: 10.1186/s40478-019-0711-9 (PMC6485101; doi:10.1186/s40478-019-0711-9)
Supplement: Supplementary file 2 — Table S2. Translation Factor screen details. (PDF 157 kb) [file 40478_2019_711_MOESM2_ESM.pdf]

Table S2: Translation factor screen details

Sources
BDSC: Bloomington *Drosophila* Stock Center
VDRC: Vienna *Drosophila* Resource Center

Color scheme						
Suppressor	Mild Suppressor	No Effect	Mild Enhancer	Enhancer	Lethal Enhancer	not tested

Fly Gene			Human	Loss-of-function (LOF) line tested					Final Call	LDS-(G4C2)$_{EXP}$		(GR)$_{36}$	Alone	Control (LacZ)	
Gene Symbol	CG #	Flybase #	orthologue	Source	Stock #	Type	Details	RNAi		Toxicity	GR-GFP levels	Toxicity	Phenotype	expression	Notes
eIF4B	CG10837	FBgn0020660	EIF4B	BDSC	57305	RNAi	Valium 20	y1 sc* v1; P{TRiP.HMS04503}attP40	Candidate RAN-translation factor	Suppressor	Suppressor	Enhancer	No Effect	No Effect	Similar results seen with VDRC stock #330010 (RNAi.VSH330010)
eIF4H1	CG4429	FBgn0262734	EIF4H	BDSC	57306	RNAi	Valium 20	y1 sc* v1; P{TRiP.HMS04504}attP40	Candidate RAN-translation factor	Suppressor	Suppressor	Enhancer	No Effect	No Effect	Similar results seen with VDRC stock #100817 (RNAi.KK108805)
eIF4E3	CG8023	FBgn0265089	EIF4E	BDSC	53880	RNAi	Valium 20	y1 v1; P{TRiP.HMJ21195}attP40	Candidate RAN-translation factor	Suppressor	Suppressor	Enhancer	No Effect	No Effect	
eIF4E4	CG10124	FBgn0035709	EIF4E	BDSC	50951	RNAi	Valium 20	y1 v1; P{TRiP.HMJ21052}attP40	Candidate RAN-translation factor	Suppressor	Suppressor	Enhancer	No Effect	No Effect	
eIF4E7	CG32859	FBgn0040368	EIF4E	BDSC	33471	mutant	EP-REV	P{EP}eIF4E7G1355 w*	Candidate RAN-translation factor	Suppressor	Suppressor	No Effect		No Effect	
eIF5B	CG10840	FBgn0026259	EIF5B	BDSC	44418	RNAi	Valium 22	y1 sc* v1; P{TRiP.GL01593}attP2/TM3, Sb1	Candidate RAN-translation factor	Suppressor	Suppressor	No Effect		Mild upregulation	
eIF5	CG9177	FBgn0030719	EIF5	BDSC	34841	RNAi	Valium 20	y1 sc* v1; P{TRiP.HMS00159}attP2	Candidate RAN-translation factor	Suppressor	Suppressor	No Effect	No Effect	No Effect	
eIF2β	CG4153	FBgn0004926	EIF2S2	BDSC	53268	RNAi	Valium 20	y1 sc* v1; P{TRiP.HMC02396}attP2/TM3,	Candidate RAN-translation factor	Mild Suppressor	Suppressor	No Effect	No Effect	No Effect	
eIF3d1 (eIF-3p66)	CG10161	FBgn0040227	EIF3D	BDSC	20072	mutant	EP-REV	y1 w67c23; P{EPgy2}eIF3d1EY05735/TM3,	Candidate RAN-translation factor	Mild Suppressor	Mild Suppressor	No Effect		No Effect	
eIF4E5	CG8277	FBgn0035823	EIF4E	BDSC	66332	RNAi	Valium 20	y1 sc* v1; P{TRiP.HMC06261}attP40	Candidate RAN-translation factor	No Effect	Suppressor	Enhancer	No Effect	No Effect	
eIF3i (eIF3-S2)	CG8882	FBgn0015834	EIF3I	BDSC	34978	RNAi	Valium 20	y1 sc* v1; P{TRiP.HMS01387}attP2	Candidate RAN-translation factor	No Effect	Mild Suppressor	No Effect		No Effect	
eIF3K	CG10306	FBgn0034654	EIF3K	BDSC	44493	RNAi	Valium 20	y1 sc* v1; P{TRiP.HMC02415}attP2	Potential RAN-translation factor	No Effect	Mild Enhancer	No Effect	No Effect	No Effect	
eIF2Bγ	CG8190	FBgn0034029	EIF2B3	BDSC	41948	RNAi	Valium 20	y1 sc* v1; P{TRiP.HMS02345}attP40	G4C2 Suppressor	Suppressor	No Effect	Enhancer			
eEF2	CG2238	FBgn0000559	EEF2	BDSC	21351	mutant	EP-REV	y1 w67c23; P{EPgy2}eEF2EY02807/CyO	G4C2 Suppressor	Suppressor	No Effect	No Effect			
eIF2Bδ	CG10315	FBgn0034858	EIF2B4	BDSC	15441	mutant	EP-REV	y1 w67c23; P{EPgy2}eIF2BδEY03558	G4C2 Suppressor	Suppressor	No Effect	No Effect			
eEF1β	CG6341	FBgn0028737	EEF1B2	BDSC	16371	mutant	EP-REV	y1 w67c23; P{EPgy2}eEF1βEY05513	G4C2 Suppressor	Suppressor	No Effect	No Effect			
eIF4G1	CG10811	FBgn0023213	EIF4G3	BDSC	33049	RNAi	Valium 20	y1 sc* v1; P{TRiP.HMS00762}attP2	GR suppressor	Suppressor	Mild Enhancer	Mild Suppressor	No Effect	No Effect	
eIF3b (eIF3-S9)	CG4878	FBgn0034237	EIF3B	BDSC	32880	RNAi	Valium 20	y1 sc* v1; P{TRiP.HMS00668}attP2	GR Enhancer	Enhancer	Enhancer	Enhancer	No Effect	No Effect	
eIF3l	CG5642	FBgn0036258	EIF3L	BDSC	50959	RNAi	Valium 20	y1 v1; P{TRiP.HMJ21061}attP40/CyO	GR Enhancer	Mild Enhancer	Mild Enhancer	Enhancer	No Effect		
eIF4E1	CG4035	FBgn0015218	EIF4E	BDSC	34096	RNAi	Valium 20	y1 sc* v1; P{TRiP.HMS00969}attP2	GR Enhancer	No Effect	Mild Enhancer	Enhancer	No Effect		
NAT1	CG3845	FBgn0010488	EIF4G2	BDSC	32357	RNAi	Valium 20	y1 sc* v1; P{TRiP.HMS00348}attP2	GR Enhancer	No Effect	Enhancer	Mild Enhancer	No Effect	No Effect	
eIF5C	CG2922	FBgn0250753	BZW1	BDSC	27248	RNAi	Valium 10	y1 v1; P{TRiP.JF02556}attP2	GR Enhancer	Enhancer	Enhancer	Enhancer	No Effect		similar results seen with BDSC stock #38918 (RNAi.TRiP.GL00690)
eIF1	CG17737	FBgn0035423	EIF1B	BDSC	57174	RNAi	Valium 20	y1 sc* v1; P{TRiP.HMC04556}attP40	No Effect	No Effect	No Effect	Enhancer			

eIF2Bα	CG7883	FBgn0039726	EIF2B1	BDSC	55624	RNAi	Valium 20	y1 sc* v1; P{TRiP.HMC03768}attP40	No Effect	No Effect	No Effect				
eIF2Bβ	CG2677	FBgn0024996	EIF2B2	BDSC	55675	RNAi	Valium 20	y1 sc* v1; P{TRiP.HMC03838}attP40	No Effect	No Effect	No Effect				
eIF2Bε	CG3806	FBgn0023512	EIF2B5	BDSC	55321	RNAi	Valium 20	y1 v1; P{TRiP.HMC04008}attP40	No Effect	No Effect	No Effect				
eIF2D	CG31426	FBgn0041588	EIF2D	BDSC	33995	RNAi	Valium 20	y1 sc* v1; P{TRiP.HMS00958}attP2	No Effect	No Effect	No Effect				
eIF3c (eIF3-S8)	CG4954	FBgn0034258	EIF3CL	BDSC	16857	mutant	EP-REV	y1 w67c23; P{EPgy2}eIF3cEY07713/CyO	No Effect	No Effect	No Effect				
eIF3f1 (eIF3-S5)	CG9769	FBgn0037270	EIF3F	BDSC	33980	RNAi	Valium 20	y1 sc* v1; P{TRiP.HMS00940}attP2	No Effect	No Effect	No Effect				
eIF3g1 (eIF3-S4)	CG8636	FBgn0029629	EIF3G	BDSC	43243	RNAi	Valium 22	y1 sc* v1; P{TRiP.GLC01430}attP2	No Effect	No Effect	No Effect				
eIF3h (eIF-3p40)	CG9124	FBgn0022023	EIF3H	BDSC	55603	RNAi	Valium 22	y1 sc* v1; P{TRiP.GL01831}attP2	No Effect	No Effect	No Effect				
eIF4AIII	CG7483	FBgn0037573	EIF4A3	BDSC	32444	RNAi	Valium 20	y1 sc* v1; P{TRiP.HMS00442}attP2	No Effect	No Effect	No Effect		No Effect		
eIF4G2	CG10192	FBgn0260634	EIF4G1, EIF4G3	BDSC	42893	RNAi	Valium 20	y1 sc* v1; P{TRiP.HMS02586}attP40	No Effect	No Effect	No Effect				
eIF4H2	CG1340	FBgn0039797	EIF4H	VDRC	v32191	RNAi	GD	w1118; P{GD8011}v32191/CyO	No Effect	No Effect	No Effect				expressed in sperm; similar results seen with VDRC stock #102825 (RNAi.KK103972)
eEF1α1	CG8280	FBgn0284245	EEF1A2	BDSC	33960	RNAi	Valium 20	y1 sc* v1; P{TRiP.HMS00917}attP2	No Effect	No Effect	No Effect				
eEF1α2	CG1873	FBgn0000557	EEF1A2	BDSC	64659	RNAi	Valium 20	y1 sc* v1; P{TRiP.HMC05694}attP40	No Effect	No Effect	No Effect				
eEF1γ	CG11901	FBgn0029176	EEF1G	BDSC	31811	mutant	EP-REV	w1118; P{EP}eEF1γG16379/TM6C, Sb1	No Effect	No Effect	No Effect	No Effect	Mild Enhancer	No Effect	
eEF1δ	CG4912	FBgn0032198	EEF1D	BDSC	29605	RNAi	Valium 10	y1 v1; P{TRiP.JF03284}attP2	No Effect	No Effect	No Effect	No Effect			
eEFSec	CG9841	FBgn0034627	EEFSEC	BDSC	42805	RNAi	Valium 22	y1 v1; P{TRiP.GL01178}attP2	No Effect	No Effect	No Effect	No Effect			
eRF1	CG5605	FBgn0036974	ETF1	BDSC	17265	mutant	EP-REV	w1118; P{EP}eRF1EP3195/TM6B, Tb1	No Effect	No Effect	No Effect				
eRF3	CG6382	FBgn0020443	GSPT1	BDSC	36703	RNAi	Valium 20	y1 sc* v1; P{TRiP.HMS01592}attP2	No Effect	No Effect	No Effect				
eIF4EHP	CG33100	FBgn0053100	EIF4E2	BDSC	43990	RNAi	Valium 20	y1 sc* v1; P{TRiP.HMS02703}attP40	No Effect	No Effect	No Effect				
eIF1A	CG8053	FBgn0026250	EIF1AY	BDSC	29316	RNAi	Valium 10	y1 v1; P{TRiP.JF02475}attP2	Unspecific enhancer	Enhancer		Enhancer	Enhancer		
eIF2α	CG9946	FBgn0261609	EIF2S1	BDSC	44449	RNAi	Valium 22	y1 v1; P{TRiP.GLC01598}attP2/TM3,	Unspecific enhancer	Enhancer	Enhancer	Enhancer	Enhancer		
eIF2γ	CG43665	FBgn0263740	EIF2S3	BDSC	33401	RNAi	Valium 20	y1 sc* v1; P{TRiP.HMS00279}attP2	Unspecific enhancer	Enhancer	Enhancer	Enhancer	Enhancer		similar results seen with BDSC stock #32914 (RNAi.TRiP.HMS00704)
eIF3a (eIF3-S10)	CG9805	FBgn0037249	EIF3A	BDSC	34353	RNAi	Valium 20	y1 sc* v1; P{TRiP.HMS01342}attP2	Unspecific enhancer	Enhancer	Enhancer	Enhancer	Mild Enhancer	No Effect	
eIF3m	CG8309	FBgn0033902	EIF3M	BDSC	32879	RNAi	Valium 20	y1 sc* v1; P{TRiP.HMS00667}attP2	Unspecific enhancer				Mild Enhancer		
eIF4A	CG9075	FBgn0001942	EIF4A1	BDSC	33970	RNAi	Valium 20	y1 sc* v1; P{TRiP.HMS00927}attP2	Unspecific enhancer				Lethal Enhancer		
eIF3d2	CG4810	FBgn0037994	EIF3D						not tested						
eIF3e (eIF3-S6)	CG9677	FBgn0025582	EIF3E						not tested						
eIF3f2	CG8335	FBgn0033069	EIF3F						not tested						
eIF3g2 (eIF3gb)	CG10881	FBgn0038796	EIF3G						not tested						
eIF3j	CG12131	FBgn0027619	EIF3J						not tested						

eIF4E6	CG1442	FBgn0039622	EIF4E, EIF4E1B						not tested						
eIF5A (eEF5)	CG3186	FBgn0285952	EIF5A2						not tested						
eIF6	CG17611	FBgn0034915	EIF6						not tested						
