# Supplementary material for: eIF4B and eIF4H mediate GR production from expanded G4C2 in a Drosophila model for C9orf72-associated ALS
Source: Acta Neuropathol Commun. 2019 Apr 25;7:62. doi: 10.1186/s40478-019-0711-9 (PMC6485101; doi:10.1186/s40478-019-0711-9)
Supplement: Supplementary file 3 — Table S3. Fibroblast lines. (PDF 56 kb) [file 40478_2019_711_MOESM3_ESM.pdf]

Table S3: cell lines

Fibroblast cell line #	NINDS/Coriell Code	Mutation	Diagnosis	Age at onset	Age at sampling	Gender	Source
1	ND29178	n/a	healthy	n/a	66	M	Rutgers
2	ND29510	n/a	healthy	n/a	55	F	Rutgers
3	ND34769	n/a	healthy	n/a	68	F	Rutgers
4	ND36320	n/a	healthy	n/a	71	F	Rutgers
5	ND38530	n/a	healthy	n/a	55	M	Rutgers
6	ND40069	*C9orf72: Intermediate*	Parkinsonism	71	75	F	Rutgers
7	ND42496	*C9orf72*	FTD	At Risk	57	M	Rutgers
8	ND42504	*C9orf72*	FTD	At Risk	53	F	Rutgers
9	ND42506	*C9orf72*	FTD	At Risk	46	F	Rutgers
