# Supplementary material for: eIF4B and eIF4H mediate GR production from expanded G4C2 in a Drosophila model for C9orf72-associated ALS
Source: Acta Neuropathol Commun. 2019 Apr 25;7:62. doi: 10.1186/s40478-019-0711-9 (PMC6485101; doi:10.1186/s40478-019-0711-9)
Supplement: Supplementary file 4 — Table S4. Patient samples. (PDF 34 kb) [file 40478_2019_711_MOESM4_ESM.pdf]

Table S4: Patient samples

The sample median (minimum, 25th percentile, 75th percentile, and maximum) is given for age at onset, age at death and disease duration, all given in years.

	***C9orf72*-positive**			
	**All**	**ALS**	**ALS/FTLD**	**FTLD**
**Number of cases**	66	19	21	26
**Gender (male)**	38 (57.58%)	7 (36.84%)	12 (57.14%)	19 (73.08%)
**Age at onset**	61 (41, 55, 67, 79)	56 (41, 49, 61, 69)	58 (50, 54, 65, 74)	66 (44, 57, 70, 79)
**Age at death**	64 (43, 59, 72, 90)	60 (43, 51, 64, 72)	62 (51, 60, 68, 80)	72 (52, 65, 78, 90)
**Disease duration**	3.9 (1.0, 2.3, 6.7, 14.5)	2.1 (1.0, 1.5, 3.1, 6.3)	4.8 (1.1, 2.7, 6.7, 10.4)	7.3 (2.4, 5.8, 9.4, 14.5)

	***C9orf72*-negative**			
	**All**	**ALS**	**ALS/FTLD**	**FTLD**
**Number of cases**	46	21	9	16
**Gender (male)**	21 (45.65%)	10 (47.62%)	3 (33.33%)	8 (50.00%)
**Age at onset**	66 (45, 57, 73, 86)	59 (45, 49, 66, 76)	70 (46, 57, 70, 78)	73 (57, 66, 80, 86)
**Age at death**	72 (47, 64, 83, 100)	66 (47, 59, 71, 83)	73 (49, 63, 78, 85)	85 (66, 78, 89, 100)
**Disease duration**	4.6 (0.6, 2.9, 8.6, 21.9)	3.4 (0.9, 2.0, 4.8, 21.5)	2.9 (0.6, 1.8, 7.8, 21.9)	8.5 (3.9, 8.2, 10.1, 20.5)

	**Healthy controls**
**Number of cases**	22
**Gender (male)**	12 (54.54%)
**Age at death**	79 (57, 64, 89, 99)
