# Supplementary material for: eIF4B and eIF4H mediate GR production from expanded G4C2 in a Drosophila model for C9orf72-associated ALS
Source: Acta Neuropathol Commun. 2019 Apr 25;7:62. doi: 10.1186/s40478-019-0711-9 (PMC6485101; doi:10.1186/s40478-019-0711-9)
Supplement: Supplementary file 5 — Table S5. Fly lines used. (PDF 80 kb) [file 40478_2019_711_MOESM5_ESM.pdf]

Table S5: Fly lines used

Name	Location	Source	ID #	Full genotype
Gmr-GAL4 (III)	3	Matthew Freeman	n/a	w*;; Gmr-GAL4^{YH3}/TM3,Sb
Gmr-GAL4 (II)	2	BDSC	1104	w*; Gmr-GAL4;
Da-GAL4	3	BDSC	55851	w*;; Da-GAL4
HS-GAL4	3	BDSC	1799	w[*];; P{w[+mC]=GAL4-Hsp70.PB}89-2-1
Control (DSRED)	2	Nancy Bonini	n/a	w[1118]; UAS-DSRED;
LDS-(G4C2)$_{CTRL}$; LDS-(G4C2)12$^{GR\text{-}GFP}$	3	Nancy Bonini	n/a	w[1118];; UAS-LDS-(G4C2)$_{4,9,12}$[GR-GFP]
LDS-(G4C2)$_{EXP}$; LDS-(G4C2)44$^{GR\text{-}GFP}$	3	Nancy Bonini	n/a	w[1118];; UAS-LDS-(G4C2)$_{4,42,44}$[GR-GFP]
Control (w^{1118})	n/a	BDSC	5905	w[1118];;
Control RNAi	3	BDSC	31603	y[1] v[1];; P{y[+t7.7] v[+t1.8]=TRiP.JF01355}attP2
(GR)36	2	BDSC	58692	w[1118]; P{y[+t7.7] w[+mC]=UAS-poly-GR.PO-36}attP40;
eIF4B RNAi	2	BDSC	57305	y1 sc* v1; P{TRiP.HMS04503}attP40;
eIF4B RNAi-2	2	VDRC	330010	w*; P{VSH330010}attP40;
eIF4H1 RNAi	2	BDSC	57306	y1 sc* v1; P{TRiP.HMS04504}attP40;
eIF4H1 RNAi-2	2	VDRC	100817	w*; P{KK108805}VIE-260B;

BDSC = Bloomington *Drosophila* Stock Center

VDRC = Vienna *Drosophila* Resource Center
