# Supplementary material for: eIF4B and eIF4H mediate GR production from expanded G4C2 in a Drosophila model for C9orf72-associated ALS
Source: Acta Neuropathol Commun. 2019 Apr 25;7:62. doi: 10.1186/s40478-019-0711-9 (PMC6485101; doi:10.1186/s40478-019-0711-9)
Supplement: Supplementary file 6 — Table S6. primers. (PDF 74 kb) [file 40478_2019_711_MOESM6_ESM.pdf]

Table S6: primers

Target	Species	Assay	Forward (5'-3')	Reverse (5'-3')	Reference
LDS-G4C2	*Dmel.*	PCR	ACTCGCTGAGGGTGAACAAG	CGACTCCTGAGTTCCAGAGC	Goodman et al, Nat Neuro, in press
GFP tag	*Dmel.*	qPCR	ACGTAAACGGCCACAAGTTC	AAGTCGTGCTGCTTCATGTG	Goodman et al, Nat Neuro, in press
RP49	*Dmel.*	qPCR	TGTCCTTCCAGCTTCAAGATGACCATC	CTTGGGCTTGCGCCATTTGTG	Gabler, M. et al., 2005
eIF4B	*Dmel.*	qPCR	CGCATTGAGCTATCGAATGA	CCAATTTCCGGAATCCCTAT	this paper
eIF4H1	*Dmel.*	qPCR	GGGAAACGGATCAGTTCAAA	CTTCTGGAAACCGTCTCTGC	this paper
EIF4H	*Hsap.*	qPCR	GGTGGCTTTGGATTCAGAAA	CCCTGAAGCCAGAATTGAAG	this paper
EIF4B	*Hsap.*	qPCR	AGCTCAGACACAGAGCAGCA	CTTTCCTTCCTGGTCCTTCC	this paper
RPLP0	*Hsap*	qPCR	TCTACAACCCTGAAGTGCTTGAT	CAATCTGCAGACAGACACTGG	Goodman et al, Nat Neuro, in press
GAPDH	*Hsap*	qPCR	GTTCGACAGTCAGCCGCATC	GGAATTTGCCATGGGTGGA	Goodman et al, Nat Neuro, in press
