# Supplementary material for: eIF4B and eIF4H mediate GR production from expanded G4C2 in a Drosophila model for C9orf72-associated ALS
Source: Acta Neuropathol Commun. 2019 Apr 25;7:62. doi: 10.1186/s40478-019-0711-9 (PMC6485101; doi:10.1186/s40478-019-0711-9)
Supplement: Supplementary file 7 — Table S1. Full genotypes in figures. (PDF 121 kb) [file 40478_2019_711_MOESM7_ESM.pdf]

Table S1: Full genotypes in figures

Fig. 1C	Genotype (driver: HS-GAL4, BL2077)	Includes	
LDS-$(G4C2)_{CTRL}^{GR-GFP}$	w^{1118}, UAS-LDS-$(G4C2)_{4,9,12}^{GR-GFP}$/w*; GAL4-Hsp70/+;		
LDS-$(G4C2)_{EXP}^{GR-GFP}$	w^{1118}/w*; GAL4-Hsp70/+; UAS-LDS-$(G4C2)_{4,42,44}^{GR-GFP}$/+		
**Fig. 1D and Sup. Fig. 1**	**Genotype (driver: GMR-$GAL4^{YH3}$)**	**Includes**	**Temp**
LDS-$(G4C2)_{CTRL}^{GR-GFP}$	w^{1118}, UAS-LDS-$(G4C2)_{4,9,12}^{GR-GFP}$/w^{1118};; GMR-$GAL4^{YH3}$/+	UAS-DSRED	26°C
LDS-$(G4C2)_{EXP}^{GR-GFP}$	w^{1118};; UAS-LDS-$(G4C2)_{4,42,44}^{GR-GFP}$/GMR-$GAL4^{YH3}$		26°C
GFP CTRL	w^{1118};; UAS-GFP.NLS/GMR-$GAL4^{YH3}$	BDSC #4776	26°C
Neg CTRL	w^{1118};; GMR-$GAL4^{YH3}$/+		26°C
**Fig. 1E**	**Genotype (driver: GMR-$GAL4^{YH3}$)**	**Includes**	**Temp**
Positive Control	w^{1118}/Y; UAS-DSRED/+; GMR-$GAL4^{YH3}$/+	UAS-DSRED	26°C
LDS-$(G4C2)_{CTRL}^{GR-GFP}$	w^{1118}, UAS-LDS-$(G4C2)_{4,9,12}^{GR-GFP}$/Y;; GMR-$GAL4^{YH3}$/+		26°C
LDS-$(G4C2)_{EXP}^{GR-GFP}$	w^{1118}/Y;; UAS-LDS-$(G4C2)_{4,42,44}^{GR-GFP}$/GMR-$GAL4^{YH3}$		26°C
**Fig. 1F**	**Genotype (driver: GMR-$GAL4^{YH3}$)**	**Includes**	**Temp**
DSRED	w^{1118}/Y; UAS-DSRED/+; GMR-$GAL4^{YH3}$/+	UAS-DSRED	26°C
LDS-$(G4C2)_{CTRL}^{GR-GFP}$	w^{1118}, UAS-LDS-$(G4C2)_{4,9,12}^{GR-GFP}$/Y;; GMR-$GAL4^{YH3}$/+		26°C
LDS-$(G4C2)_{EXP}^{GR-GFP}$	w^{1118}/Y;; UAS-LDS-$(G4C2)_{4,42,44}^{GR-GFP}$/GMR-$GAL4^{YH3}$		26°C
**Fig. 2A - external eye toxicity**	**Genotype (driver: GMR-$GAL4^{YH3}$)**	**Includes**	**Temp**
LDS-$(G4C2)_{EXP}^{GR-GFP}$ w/ eIF4B RNAi	w^{1118}/Y; UAS-eIF4B $RNAi^{HMS04503}$/+; UAS-LDS-$(G4C2)_{4,42,44}^{GR-GFP}$, GMR-$GAL4^{YH3}$/+	BDSC #57305	26°C
LDS-$(G4C2)_{EXP}^{GR-GFP}$ w/ Control RNAi	w^{1118}/Y;; UAS-LDS-$(G4C2)_{4,42,44}^{GR-GFP}$, GMR-$GAL4^{YH3}$/UAS-Luc $RNAi^{JF01355}$	BDSC #31603	26°C
LDS-$(G4C2)_{EXP}^{GR-GFP}$ w/ eIF3B RNAi	w^{1118}/Y;; UAS-LDS-$(G4C2)_{4,42,44}^{GR-GFP}$, GMR-$GAL4^{YH3}$/UAS-eIF3B $RNAi^{HMS00668}$	BDSC #32880	26°C
$(GR)_{36}$ w/ eIF4G1 RNAi	w^{1118}/y^{1}, sc*, v^{1}; UAS-$(GR)_{36}$/+; GMR-$GAL4^{YH3}$/UAS-eIF4G1 $RNAi^{HMS00762}$	BDSC #33049	24°C
$(GR)_{36}$ w/ Control RNAi	w^{1118}/y^{1}, v^{1}; UAS-$(GR)_{36}$/+; GMR-$GAL4^{YH3}$/UAS-Luc $RNAi^{JF01355}$	BDSC #31603	24°C
$(GR)_{36}$ w/ eIF4B RNAi	w^{1118}/y^{1}, sc*, v^{1}; UAS-$(GR)_{36}$/UAS-eIF4B $RNAi^{HMS04503}$; GMR-$GAL4^{YH3}$/+	BDSC #57305	24°C
**Fig. 2A - external eye GR-GFP**	**Genotype (driver: GMR-$GAL4^{YH3}$)**	**Includes**	**Temp**
LDS-$(G4C2)_{EXP}^{GR-GFP}$ w/ eIF4B RNAi	w^{1118}/y^{1}, sc*, v^{1}; UAS-eIF4B $RNAi^{HMS04503}$/+; UAS-LDS-$(G4C2)_{4,42,44}^{GR-GFP}$, GMR-$GAL4^{YH3}$/+	BDSC #57305	26°C
LDS-$(G4C2)_{EXP}^{GR-GFP}$ w/ Control RNAi	w^{1118}/y^{1}, v^{1};; UAS-LDS-$(G4C2)_{4,42,44}^{GR-GFP}$, GMR-$GAL4^{YH3}$/UAS-Luc $RNAi^{JF01355}$	BDSC #31603	26°C
LDS-$(G4C2)_{EXP}^{GR-GFP}$ w/ eIF3B RNAi	w^{1118}/y^{1}, sc*, v^{1};; UAS-LDS-$(G4C2)_{4,42,44}^{GR-GFP}$, GMR-$GAL4^{YH3}$/UAS-eIF3B $RNAi^{HMS00668}$	BDSC #32880	26°C
**Fig. 3A**	**Genotype (driver: Da-GAL4, III)**	**Includes**	**Temp**

Control RNAi	*w^{1118}/Y;; UAS-Luc RNAi^{JF01355}/Da-GAL4*	BDSC #31603	26˚C
eIF4B RNAi	*w^{1118}/Y; UAS-eIF4B RNAi^{HMS04503}/+; Da-GAL4/+*	BDSC #57305	26˚C
eIF4H1 RNAi	*w^{1118}/Y; UAS-eIF4H1 RNAi^{HMS04504}/+; Da-GAL4/+*	BDSC #57306	26˚C
**Fig. 3B**	**Genotype (driver: Da-GAL4, III)**	**Includes**	**Temp**
progeny with eIF4B RNAi	*UAS-eIF4B RNAi^{HMS04503}/+; Da-GAL4/+*	BDSC #57305	26˚C
progeny without eIF4B RNAi	*CyO/+; Da-GAL4/+*		26˚C
progeny with eIF4H1 RNAi	*UAS-eIF4B RNAi^{HMS04504}/+; Da-GAL4/+*	BDSC #57306	26˚C
progeny without eIF4H1 RNAi	*CyO/+; Da-GAL4/+*		26˚C
**Fig. 4A-B and 5A-B**	**Genotype (driver: GMR-GAL4^{YH3})**	**Includes**	**Temp**
LDS-(G4C2)$_{EXP}^{GR-GFP}$ w/ Control RNAi	*w^{1118}/Y;; UAS-LDS-(G4C2)$_{4,42,44}^{GR-GFP}$, GMR-GAL4^{YH3}/UAS-Luc RNAi^{JF01355}*	BDSC #31603	26˚C
LDS-(G4C2)$_{EXP}^{GR-GFP}$ w/ eIF4B RNAi	*w^{1118}/Y; UAS-eIF4B RNAi^{HMS04503}/+; UAS-LDS-(G4C2)$_{4,42,44}^{GR-GFP}$, GMR-GAL4^{YH3}/+*	BDSC #57305	26˚C
LDS-(G4C2)$_{EXP}^{GR-GFP}$ w/ eIF4H1 RNAi	*w^{1118}/Y; UAS-eIF4H1 RNAi^{HMS04504}/+; UAS-LDS-(G4C2)$_{4,42,44}^{GR-GFP}$, GMR-GAL4^{YH3}/+*	BDSC #57306	26˚C
**Fig. 4C-D**	**Genotype (driver: GMR-GAL4^{YH3})**	**Includes**	**Temp**
(GR)$_{36}$ w/ Control RNAi	*w^{1118}/y^{1}, v^{1}; UAS-(GR)$_{36}$/+; GMR-GAL4^{YH3}/UAS-Luc RNAi^{JF01355}*	BDSC #31603	24˚C
(GR)$_{36}$ w/ eIF4B RNAi	*w^{1118}/y^{1}, sc*, v^{1}; UAS-(GR)$_{36}$/UAS-eIF4B RNAi^{HMS04503}; GMR-GAL4^{YH3}/+*	BDSC #57305	24˚C
(GR)$_{36}$ w/ eIF4B RNAi	*w^{1118}/y^{1}, sc*, v^{1}; UAS-(GR)$_{36}$/UAS-eIF4B RNAi^{HMS04504}; GMR-GAL4^{YH3}/+*	BDSC #57306	24˚C
**Fig. 4E-F**	**Genotype (driver: GMR-GAL4^{YH3})**	**Includes**	**Temp**
Control RNAi	*w^{1118}/Y;; GMR-GAL4^{YH3}/UAS-Luc RNAi^{JF01355}*	BDSC #31603	26˚C
eIF4B RNAi	*w^{1118}/Y; UAS-eIF4B RNAi^{HMS04503}/+; GMR-GAL4^{YH3}/+*	BDSC #57305	26˚C
eIF4H1 RNAi	*w^{1118}/Y; UAS-eIF4H1 RNAi^{HMS04504}/+; GMR-GAL4^{YH3}/+*	BDSC #57306	26˚C
**Fig. 5C-D**	**Genotype (driver: GMR-GAL4^{1104})**	**Includes**	**Temp**
DsRed w/ Control RNAi	*w^{1118}/Y; UAS-DsRed, GMR-GAL4/+; UAS-Luc RNAi^{JF01355}/+*	BDSC #31603	26˚C
DsRed w/ eIF4B RNAi	*w^{1118}/Y, v^{1}; UAS-DsRed, GMR-GAL4/UAS-eIF4B RNAi^{HMS04503};*	BDSC #57305	26˚C
DsRed w/ eIF4B RNAi	*w^{1118}/Y; UAS-DsRed, GMR-GAL4/UAS-eIF4B RNAi^{HMS04504};*	BDSC #57306	26˚C
**Fig. 5E**	**Genotype (driver: GMR-GAL4^{1104})**	**Includes**	**Temp**
LacZ w/ Control RNAi	*w^{1118}/Y; UAS-LacZ, GMR-GAL4/+; UAS-Luc RNAi^{JF01355}/+*	BDSC #31603	26˚C
LacZ w/ eIF4B RNAi	*w^{1118}/Y, v^{1}; UAS-LacZ, GMR-GAL4/UAS-eIF4B RNAi^{HMS04503};*	BDSC #57305	26˚C
LacZ w/ eIF4B RNAi	*w^{1118}/Y; UAS-LacZ, GMR-GAL4/UAS-eIF4B RNAi^{HMS04504};*	BDSC #57306	26˚C
**Sup. Fig. 2**	**Genotype (driver: GMR-GAL4^{YH3})**	**Includes**	**Temp**
Control RNAi	*w^{1118}/Y;; UAS-LDS-(G4C2)$_{4,42,44}^{GR-GFP}$, GMR-GAL4^{YH3}/UAS-Luc RNAi^{JF01355}*	BDSC #31603	26˚C

eIF5B RNAi	*w*^{1118} */Y;; UAS-LDS-(G4C2)*$_{4,42,44}^{GR\text{-}GFP}$*, GMR-GAL4*^{YH3} */UAS-eIF5B RNAi*^{GL01593}	BDSC #57305	26°C
eIF5 RNAi	*w*^{1118} */Y;; UAS-LDS-(G4C2)*$_{4,42,44}^{GR\text{-}GFP}$*, GMR-GAL4*^{YH3} */UAS-eIF5 RNAi*^{HMS00159}	BDSC #34841	26°C
eIF2β RNAi	*w*^{1118} */Y;; UAS-LDS-(G4C2)*$_{4,42,44}^{GR\text{-}GFP}$*, GMR-GAL4*^{YH3} */UAS-eIF2β RNAi*^{HMC02396}	BDSC #53268	26°C
eIF2Bα RNAi	*w*^{1118} */Y; UAS-eIF2Bα RNAi*^{HMC03768} */+; UAS-LDS-(G4C2)*$_{4,42,44}^{GR\text{-}GFP}$*, GMR-GAL4*^{YH3} */+*	BDSC #55624	26°C
eIF3g1 RNAi	*w*^{1118} */Y;; UAS-LDS-(G4C2)*$_{4,42,44}^{GR\text{-}GFP}$*, GMR-GAL4*^{YH3} */UAS-eIF3g1 RNAi*^{GLC01430}	BDSC #43243	26°C
eEFSec RNAi	*w*^{1118} */Y;; UAS-LDS-(G4C2)*$_{4,42,44}^{GR\text{-}GFP}$*, GMR-GAL4*^{YH3} */UAS-eIF3g1 RNAi*^{GL01178}	BDSC #42805	26°C
**Sup. Fig. 3A**	**Genotype (driver: Da-GAL4, III)**	**Includes**	**Temp**
w^{1118}	*w*^{1118} */Y;; Da-GAL4/+*		26°C
eIF4B RNAi-2	*w*^{1118} */Y; UAS-eIF4B RNAi*^{330010} */+; Da-GAL4/+*	VDRC #330010	26°C
eIF4H1 RNAi-2	*w*^{1118} */Y; UAS-eIF4H1 RNAi*^{108805} */+; Da-GAL4/+*	VDRC #100817	26°C
**Sup Fig. 3B-E**	**Genotype (driver: GMR-GAL4^{YH3})**	**Includes**	**Temp**
Control RNAi	*w*^{1118} */Y;; UAS-LDS-(G4C2)*$_{4,42,44}^{GR\text{-}GFP}$*, GMR-GAL4*^{YH3} */UAS-Luc RNAi*^{JF01355}	BDSC #31603	26°C
eIF4B RNAi-2	*w*^{1118} */Y; UAS-eIF4B RNAi*^{330010} */+; UAS-LDS-(G4C2)*$_{4,42,44}^{GR\text{-}GFP}$*, GMR-GAL4*^{YH3} */+*	VDRC #330010	26°C
eIF4H1 RNAi-2	*w*^{1118} */Y; UAS-eIF4H1 RNAi*^{108805} */+; UAS-LDS-(G4C2)*$_{4,42,44}^{GR\text{-}GFP}$*, GMR-GAL4*^{YH3} */+*	VDRC #100817	26°C
**Sup. Fig. 3F**	**Genotype (driver: GMR-GAL4^{1104})**	**Includes**	**Temp**
LacZ w/ Control	*w*^{1118} */Y; UAS-LacZ, GMR-GAL4/UAS-DSRED;*	UAS-DSRED	26°C
LacZ w/ eIF4B RNAi-2	*w*^{1118} */Y, v*^{1} *; UAS-LacZ, GMR-GAL4/UAS-eIF4B RNAi*^{330010} *;*	VDRC #330010	26°C
LacZ w/ eIF4B RNAi-2	*w*^{1118} */Y; UAS-LacZ, GMR-GAL4/UAS-eIF4B RNAi*^{1088057} *;*	VDRC #100817	26°C
